# Semi‐Artificial Photosynthesis Enables Light‐Driven Biomanufacturing in Non‐Photosynthetic Bacteria

**DOI:** 10.1002/advs.78265

**Published:** 2026-10-14

**Authors:** Jie Zheng, Anqi Tong, Wenxin Li, Meng Qiao, Yang Gu, Muhammad Sohail, Shan He, Sheng Yang, Xing Zhang

**Affiliations:** ^1^ State Key Laboratory of Microbial Technology School of Food Science and Pharmaceutical Engineering Jiangsu Key Laboratory for Peptide Biosynthesis Nanjing Normal University Nanjing China; ^2^ Research Center of Pharmaceutical and Synthetic Biology/Department of Food Nutrition and Safety College of Engineering China Pharmaceutical University Nanjing China; ^3^ Key Laboratory of Synthetic Biology CAS Center for Excellence in Molecular Plant Sciences Chinese Academy of Sciences Shanghai China; ^4^ Faculty of Science Technology and Engineering Charles Darwin University Northern Territory Australia

**Keywords:** bacillus subtilis, cadmium sulfide, light‐driven biomanufacturing, semi‐artificial photosynthesis, semiconductor biohybrid

## Abstract

Semiconductor biohybrid systems integrate microbial biosynthesis with semiconductor light harvesting to sustainably power energy‐intensive chemical production. Although such systems have been widely explored in photosynthetic bacteria, extending them to non‐photosynthetic biomanufacturing chassis such as *Bacillus subtilis* (*B. subtilis*) remains substantially more challenging, owing to peptidoglycan‐induced nanoparticle aggregation and mass‐transfer limitations, cytotoxicity, and inefficient interfacial electron transfer from loose adsorption between inorganic semiconductors and cells. Here, we propose a non‐invasive enhancement strategy that leverages the endogenous hydrogen sulfide (H_2_S) pathway of the *B. subtilis* host and couples it with semiconductor materials to construct a semi‐artificial photosynthetic biohybrid system. This system harnesses light energy to enhance endogenous nicotinamide adenine dinucleotide phosphate hydrogen (NADPH) regeneration, thereby augmenting microbial production without genetic modification of the target biosynthetic pathways. Applied to engineered *B. subtilis* for 2’‐fucosyl lactose (2’‐FL) and surfactin production, the biohybrid system increases product titres by two‐ to three‐ fold. This strategy, integrated with metabolic engineering, is effective for rapid and enhanced chemical synthesis in *B. subtilis*, with potential applicability to other non‐photosynthetic bacteria harboring endogenous H_2_S pathways.

## Graphical abstract

A non‐invasive strategy exploits endogenous H_2_S metabolism in *B. subtilis* to drive in situ CdS biomineralization, enabling tight bio‐abiotic contact without genetic modification. Light harvested by the biomineralized nanoparticles powers NADPH regeneration, elevating 2’‐fucosyllactose and surfactin titres two‐ to three‐fold. This modular platform readily extends to other non‐photosynthetic bacteria harboring H_2_S pathways.

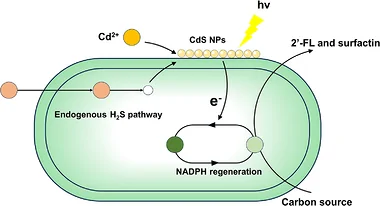

## 1 Introduction

Bacillus subtilis, a Gram‐positive model organism, is a cornerstone chassis in synthetic biology owing to its well‐characterized genetics, high secretory capacity, and engineering versatility [1]. Because reducing power, primarily NADPH, sets the flux ceiling for reductive biosynthesis, its engineering has shifted from an auxiliary consideration to a central strategy in constructing high‐performance microbial cell factories. Current redox‐centric metabolic engineering strategies in *B. subtilis* primarily involve: (i) strengthening endogenous NADPH‐generating pathways, particularly the oxidative pentose phosphate pathway (PPP), and introducing heterologous cofactor regeneration modules such as nicotinamide adenine dinucleotide (NADH) kinase (e.g., *Pos5P*) to establish efficient NADPH recycling loops, thereby enhancing intracellular reducing power availability [2, 3, 4, 5]; (ii) systematic reconstruction of central carbon metabolism through elimination of competing pathways and introduction of alternative or non‐NAD(P)^+^‐dependent routes to redistribute reducing equivalents and rebalance intracellular redox fluxes [6, 7]; and (iii) global rewiring of central carbon metabolism via combinatorial metabolic engineering, genome editing, or dynamic regulatory systems to coordinate multi‐gene expression and modulate key nodes in NADPH‐supplying pathways, ultimately optimizing redox homeostasis and biosynthetic efficiency (Figure 1a) [8, 9, 10].

**FIGURE 1:**
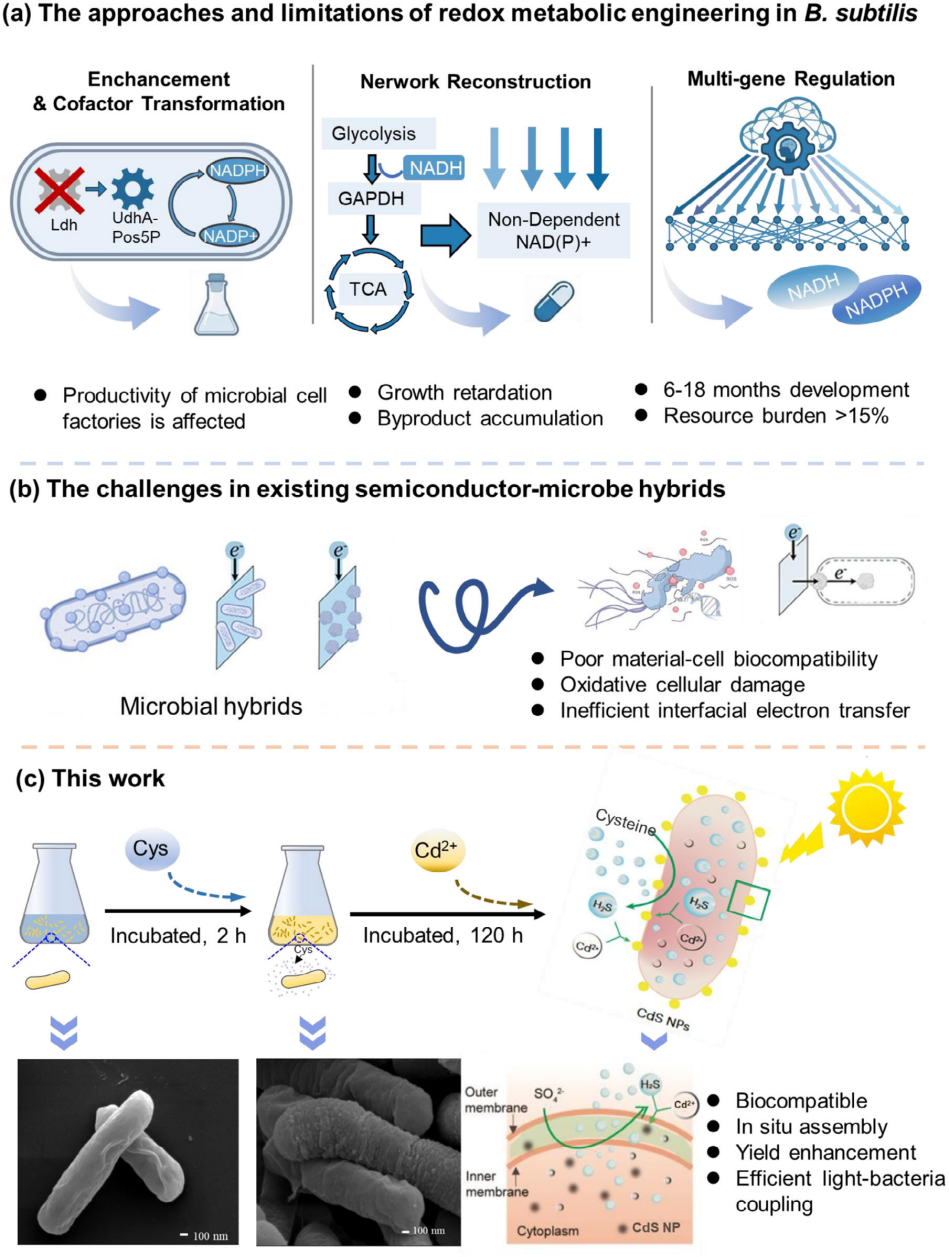
Toward an in situ self‐assembled semiconductor biohybrid for solar‐driven biosynthesis. (a) Schematic diagram showing the approaches and limitations of redox metabolic engineering in *B. subtilis*, including metabolic burden and long development cycles. (b) Schematic diagram illustrating the challenges in existing semiconductor‐microbe hybrids, such as poor adhesion and biocompatibility. (c) Schematic diagram of this work demonstrating the solar‐driven chemical enhancement in non‐photosynthetic *B. subtilis* via a semiconductor biohybrid.

Despite these advances, several inherent limitations remain. Development cycles are protracted, often requiring 6 to 18 months to optimize a single product pathway due to iterative promoter screening, ribosome binding site debugging, and genetic circuit tuning [11, 12, 13, 14, 15]. Moreover, these approaches impose a significant metabolic burden, as heterologous gene expression can account for more than 15% of cellular resources, leading to growth retardation and byproduct accumulation [16, 17, 18, 19, 20, 21, 22, 23, 24]. For instance, achieving a multi‐fold increase in surfactin titer typically demands systematic, resource‐intensive engineering that spans from the identification of bottlenecks via omics or mutagenesis to the multi‐round optimization of modules responsible for precursor supply, synthase activity, transport, and global regulation [25, 26, 27].

These challenges have driven interest in non‐genetic enhancement strategies [28], notably photocatalytic biohybrid systems that couple light‐harvesting materials with whole‐cell catalysts to supply photoexcited electrons for metabolic redox reactions. Organic semiconductors have emerged as a promising platform for such systems, leveraging tunable optoelectronic properties and inherent biocompatibility to enhance microbial electron transfer and metabolic functions. Nevertheless, critical bottlenecks must be resolved, including low electron transfer efficiency, and inadequate long‐term stability. These challenges are further exacerbated when exogenous inorganic materials are employed, as they remain intrinsically incompatible with the highly organized intracellular redox network. Poor interfacial biocompatibility leads to reduced cell viability [29, 30, 31, 32], while nonspecific and weak material‐membrane interactions (e.g., electrostatic adsorption) cause unstable adhesion, inefficient electron transfer, and substantial energy loss (Figure 1b) [29, 30, 33, 34]. Moreover, photoactive materials such as titanium dioxide can generate reactive oxygen species under illumination, inducing protein denaturation, DNA damage, and collapse of engineered metabolic networks [31, 33, 35, 36]. Collectively, these limitations arise because conventional physical blending strategies neglect the chemical specificity and spatial organization intrinsic to cellular redox regulation.

Cellular metabolism and signaling are governed by redox equilibria. Reactive sulfur species (RSS), particularly H_2_S, function as central signaling molecules alongside reactive oxygen and nitrogen species [3], regulating gene expression, metabolic flux, and stress responses via reversible cysteine modifications. This inherent biological relevance of sulfur redox chemistry motivates a fundamentally different approach to constructing semiconductor biohybrid systems. Conventional methods rely on the exogenous addition of pre‐synthesized nanoparticles, which often suffer from poor biocompatibility and unstable interfaces. In contrast, leveraging the microorganism's own metabolic pathways for the in situ synthesis of semiconductor materials offers a compelling alternative. Through endogenous sulfur metabolism‐driven biomineralization, semiconductor nanoparticles are generated directly in the cell surface, establishing intimate and stable interfacial contact with the cellular membrane.

We developed an in situ self‐assembled biohybrid system in *B. subtilis* that exploits endogenous H_2_S metabolism for interfacial semiconductor synthesis (Figure 1c). Cadmium sulfide (CdS) was selected as the light‐harvesting semiconductor for its visible‐light absorption and suitably negative conduction band. The bacterium's native cysteine metabolism generates H_2_S as an endogenous sulfur donor for in situ CdS formation. The reaction between metabolically produced H_2_S and Cd^2+^ proceeds under physiological conditions, avoiding the need for externally supplied toxic sulfide precursors and yielding nanoparticles in intimate contact with the surface of *B. subtilis*, thereby achieving biocompatible integration [37, 38, 39]. Beyond interfacial compatibility, this light‐driven biohybrid addresses a fundamental constraint in microbial fermentation. The carbon substrate typically serves a dual role as both carbon source and energy source for cofactor regeneration, creating an inherent trade‐off that limits theoretical product yield. Light energy, harnessed through in situ–formed CdS nanoparticles, decouples energy supply from carbon metabolism by providing exogenous reducing power via photogenerated electrons. This channels more carbon toward product biosynthesis while offering a renewable energy input consistent with biomanufacturing.

## 2 Result

### 2.1 Design and Preparation of *B. Subtilis*@CdS Semiconductor Biohybrid

#### 2.1.1 Endogenous H_2_S Metabolism Mediates Biohybrid Formation

H_2_S is a highly lipophilic molecule that freely diffuses across the cell membrane. The biosynthesis of H_2_S represents a pivotal node in the microbial sulfur cycle and redox regulatory network [40, 41]. As a model Gram‐positive bacterium, *B. Subtilis* possesses endogenous H_2_S metabolic pathways [42]. To verify its H_2_S‐producing capacity, cultures was supplemented with exogenous L‐cysteine, and H_2_S generation was detected using lead acetate test strips [43]. Strips positioned above *B. subtilis* cultures supplemented with L‐cysteine exhibited a pronounced brown‐to‐black color change, whereas those above unsupplemented cultures showed no discernible change (Figure 2a). H_2_S production was subsequently quantified. Upon the addition of L‐cysteine, H_2_S was generated and gradually accumulated. Notably, the presence of Cd^2^^+^ did not significantly affect H_2_S production. However, as incubation progressed and L‐cysteine became depleted, H_2_S generation ceased. The H_2_S already produced partially dissipated into the gas phase and partially reacted with Cd^2^^+^ to form CdS, ultimately leading to a decline of H_2_S concentration (Figure 2b).

**FIGURE 2:**
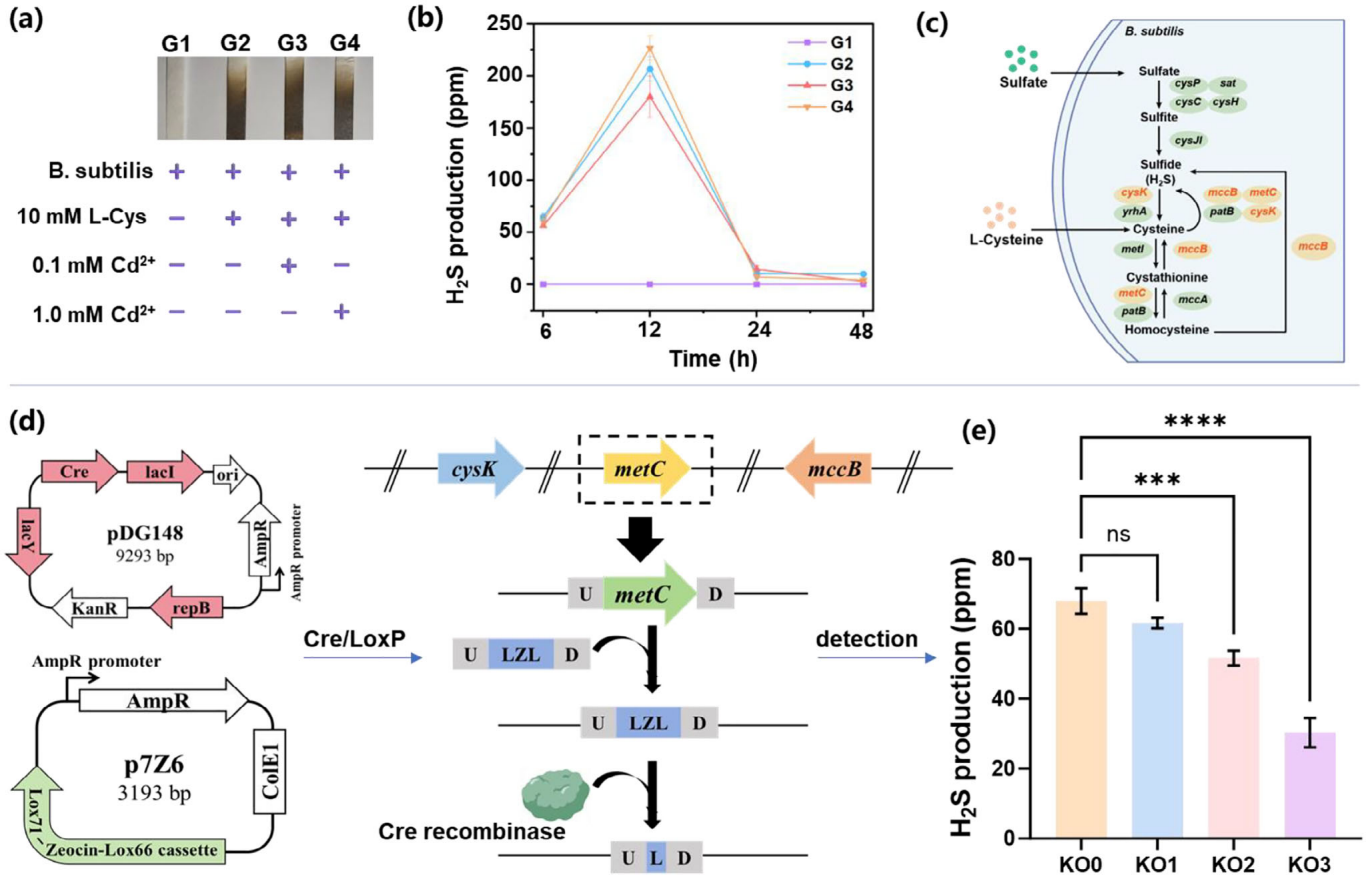
Harnessing endogenous H_2_S Metabolism in *B. subtilis* for CdS biomineralization. (a) Qualitative verification of H_2_S production induced by exogenous L‐cysteine supplementation using lead acetate test strips; (b) H_2_S production between *B. Subtilis* and *B. Subtilis*@CdS. (c) H_2_S synthesis pathway in *B. subtilis*. (d) *B. subtilis* mutants was constructed by sequentially knocking out three key enzymes (*mccB*, *metC*, and *cysK*) involved in H_2_S generation using the allelic (double‐crossover) exchange strategy. (e) Impact of knocking out key H_2_S‐synthesizing genes and the cumulative effect on H_2_S production. *n* = 3 biological replicates; One‐way ANOVA. ^***^ *p* < 0.001, ^****^ *p* < 0.0001; The bars represent the mean±S.D.

To experimentally validate the critical role of the endogenous H_2_S metabolic pathway in the formation of CdS on the bacterial surface, a series of *B. subtilis* mutants was constructed by sequentially knocking out three key enzymes (*mccB*, *metC*, and *cysK*) involved in H_2_S generation using the allelic (double‐crossover) exchange strategy (Figure 2d and Figure S1). H_2_S production in the single‐knockout mutant KO1 (*ΔmccB*) decreased by 9% compared to the non‐knockout strain KO0, while the triple‐knockout mutant KO3(*ΔmccB‐ΔmetC‐ΔcysK*) exhibited an approximately 55% reduction. These results demonstrate that H_2_S production decreased progressively with an increasing number of deleted genes, indicating a cumulative effect on H_2_S metabolic flux (Figure 2e). The sulfur metabolism network of *B. subtilis* exhibits significant functional redundancy. For example, YrhA possesses *O*‐acetylserine sulfhydrylase activity comparable to that of CysK, while PatB exhibits cystathionine β‐lyase activity analogous to that of MetC. These enzymes can partially substitute for the deleted functions, therefore sustaining H_2_S production in the KO3 (Figure 2c). SEM analysis revealed that the KO3 mutant, which produces reduced levels of H_2_S, exhibited noticeably fewer surface deposits compared to KO0, which displayed a prominent particle coating. These observations are consistent with the expectation that decreased H_2_S production leads to reduced CdS formation on the cell surface (Figure S2).

#### 2.1.2 Assembly and Characterization of *B. Subtilis*@CdS Biohybrid

Building on the validated endogenous H_2_S metabolism, we leveraged this pathway to construct *B. subtilis*@CdS biohybrids (Figure 3a). Through systematic optimization of Cd^2^^+^ and L‐cysteine concentrations, we identified that 1.0 mM Cd^2^^+^ combined with 10  mM L‐cysteine provided the optimal balance between cellular viability and 2’‐fucosyllactose (2’‐FL) production (Figures S3 and S4). Successful fabrication of semi‐biohybrids was confirmed through multi‐modal characterization. Scanning electron microscopy revealed smooth particle‐free control cells, contrasting sharply with dense coatings of CdS nanoparticles synthesized in situ on *B. subtilis* surfaces (Figure 3b,c), thereby demonstrating semiconductor nanoparticle packing. Negative staining TEM results revealed that *B. subtilis* exhibited a smooth surface with clear and intact cellular outlines. In the *B. subtilis*@CdS composite, numerous electron‐dense granular materials were observed attached to the bacterial surface with no obvious cell wall damage or morphological distortion observed, indicating that the binding of CdS particles did not significantly compromise the structural integrity of the cells (Figure 3d,e). We speculated that the intracellularly produced H_2_S, a high‐membrane‐permeability gas, readily diffused to the cell surface where it reacted with Cd^2+^ to form CdS nanoparticles. Therefore, the CdS were mainly distributed on the cell surface of the strain. In addition, we analyzed the distribution of cadmium in the culture system (Figure S5), the result supports the in situ formation of CdS on the bacterial cell surface rather than remaining in the culture medium. The measured Cd content was used to calculate the corresponding CdS mass based on the stoichiometric ratio (CdS: Cd = 144.46:112.41, w/w), and the CdS loading on *B. subtilis* was determined to be 28.60 mg CdS / g CDW (cell dry weight). We evaluated the stability of *B. subtilis*@CdS. Only minimal amounts of Cd^2+^ and CdS were released into the culture medium, indicating that the *B. subtilis*@CdS biohybrid remains highly stable (Figure S6).

**FIGURE 3:**
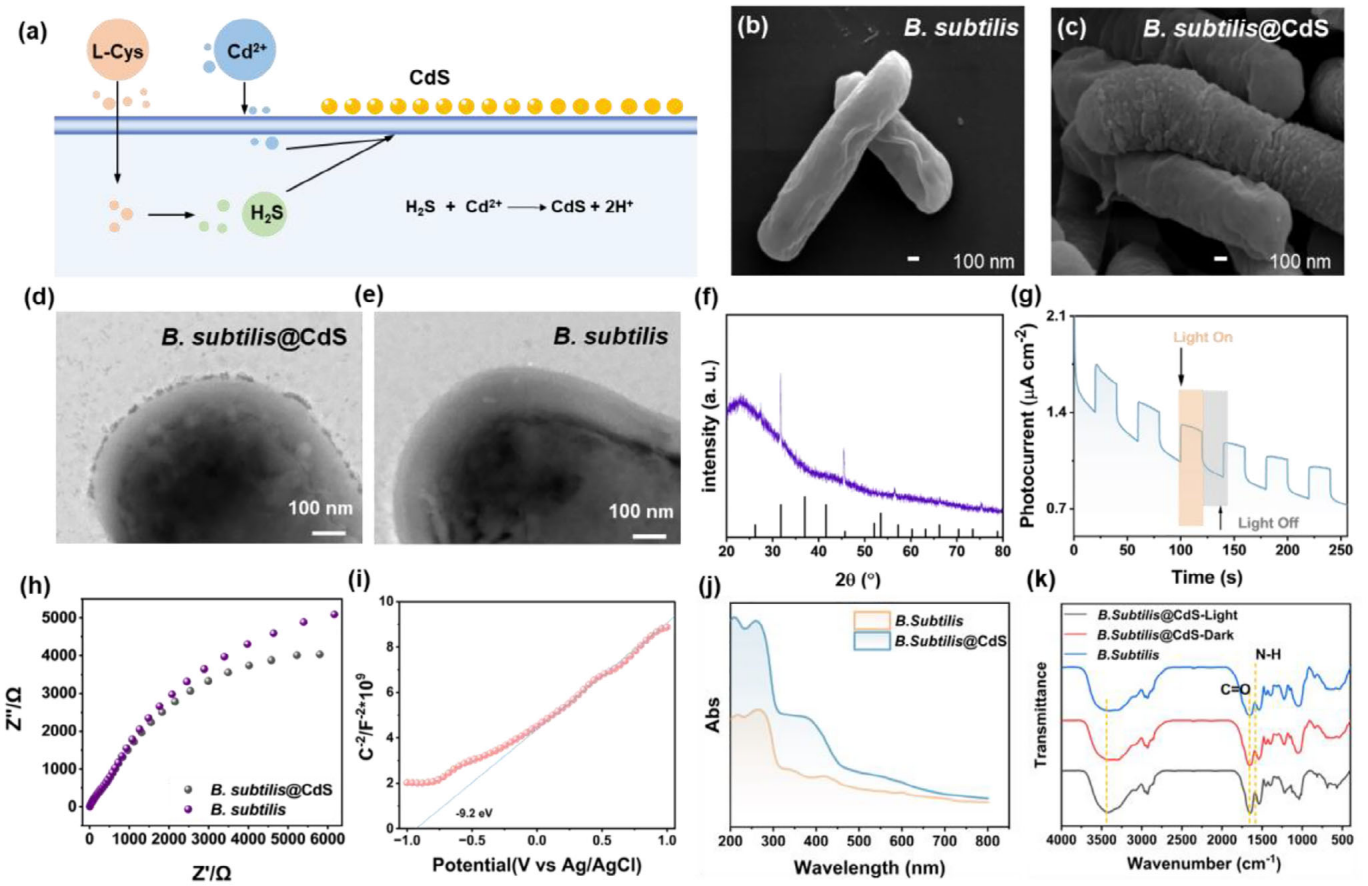
Structural, compositional, and photoelectrochemical characterization of the *B. subtilis*@CdS biohybrids. (a) Cd^2+^ and L‐cysteine could be co‐utilized by *B. subtilis* to construct a biohybrid system. (b‐c) Scanning electron microscopy (SEM) images of *B. subtilis*, and *B. subtilis*@CdS. (d‐e) Negative staining TEM of *B. Subtilis* and *B. Subtilis*@CdS. (f) X‐ray diffraction (XRD) pattern of the *B. subtilis*@CdS composite, revealing characteristic diffraction peaks corresponding to crystalline CdS. (g) Representative photocurrent response of the *B. subtilis*@CdS biohybrid under intermittent light on/off cycles (20 s intervals, 100 mW cm^−2^). (h) Electrochemical impedance spectroscopy Nyquist plot of the biohybrid, indicating reduced charge transfer resistance compared to the control. (i) Mott–Schottky plot of the *B. subtilis*@CdS. (j) UV–vis diffuse reflectance spectrum of *B. subtilis*@CdS, showing enhanced light absorption in the visible region. (k) Fourier transform infrared spectrum of *B. subtilis*@CdS, indicating the presence of characteristic functional groups associated with bacterial surface biomolecules and CdS formation.

XRD analysis of lyophilized hybrid powders definitively identified the crystalline phase as CdS (PDF 43–0985) (Figure 3f). Photoelectrochemical measurements further showed a photocurrent density of ∼0.26 µA/cm^2^ (Figure 3g). EIS Nyquist plot showed a smaller arc radius for the biohybrid compared to *B. subtilis* alone (Figure 3h), reflecting reduced electron transfer resistance. The Mott–Schottky plot of the *B. subtilis*@CdS composite electrode exhibited a positive slope, confirming its *n*‐type semiconductor characteristics. The flat band potential was determined to be −0.92 eV (Figure 3i). These values align with established CdS optoelectronic properties, confirming functional nanoparticle integration. In addition, compared with *B. subtilis*, the *B. subtilis*@CdS exhibits a characteristic absorption peak around 400 nm and extends its absorption into the visible region (Figure 3j). CdS nanoparticles on the cell surface absorb light, generating electron‐hole pairs. Photoexcited electrons from CdS reduce riboflavin shuttles secreted by *B. subtilis* itself (mediated electron transfer pathway). Reduced riboflavin shuttles transfer electrons to the membrane‐associated respiratory chain via the NADH dehydrogenase and quinone pool pathway. In the fourier Transform infrared spectroscopy (FT‐IR) spectrum, the broad band at 3000–3500 cm^−^^1^ (O–H and N–H stretching) showed modest broadening in the *B. subtilis*@CdS biohybrid compared with pristine *B. subtilis*, possibly reflecting perturbations in hydrogen bonding or coordinating interactions with the CdS surface. In the 1600–1700 cm^−^^1^ region (Amide I/II), a subtle attenuation of the Amide II band was observed, consistent with the possible involvement of surface proteinaceous structures in CdS binding, though physical shielding by the CdS layer may also contribute (Figure 3k).

To comprehensively characterize the CdS formed within the semiconductor biohybrids, the nanoparticles were isolated for detailed structural and compositional analyses. Transmission electron microscope (TEM) revealed that the isolated CdS nanoparticles obtained after cell lysis formed dense aggregate and STEM–energy‐dispersive x‐ray spectroscopy (STEM‐EDS) mapping showed that the biohybrids were composed of Cd and S elements with a high degree of spatial colocalization (Figure 4a, Figure S7), while XRD further confirmed that the isolated particles were CdS (Figure 4b). EIS data verified that the as‐prepared CdS nanoparticles exhibited good photoelectrochemical response and interfacial ion transport properties (Figure 4c). The flat‐band potential was approximately −0.72 eV (Figure 4d). These values align with established CdS optoelectronic properties, confirming functional nanoparticle integration. The nanoparticles exhibited high crystallinity, with primary lattice spacings of 3.36 and 2.05 Å, consistent with the XRD‐identified CdS phase (Figure 4e). STEM analysis revealed an average particle size of ∼5.54 nm, comparable to previously reported biogenic nanoparticles. High‐angle annular dark‐field STEM (HAADF–STEM) and selected area electron diffraction (SAED) analyses of lysed materials demonstrated densely polycrystalline CdS packing on bacterial surfaces (Figure 4f,g).

**FIGURE 4:**
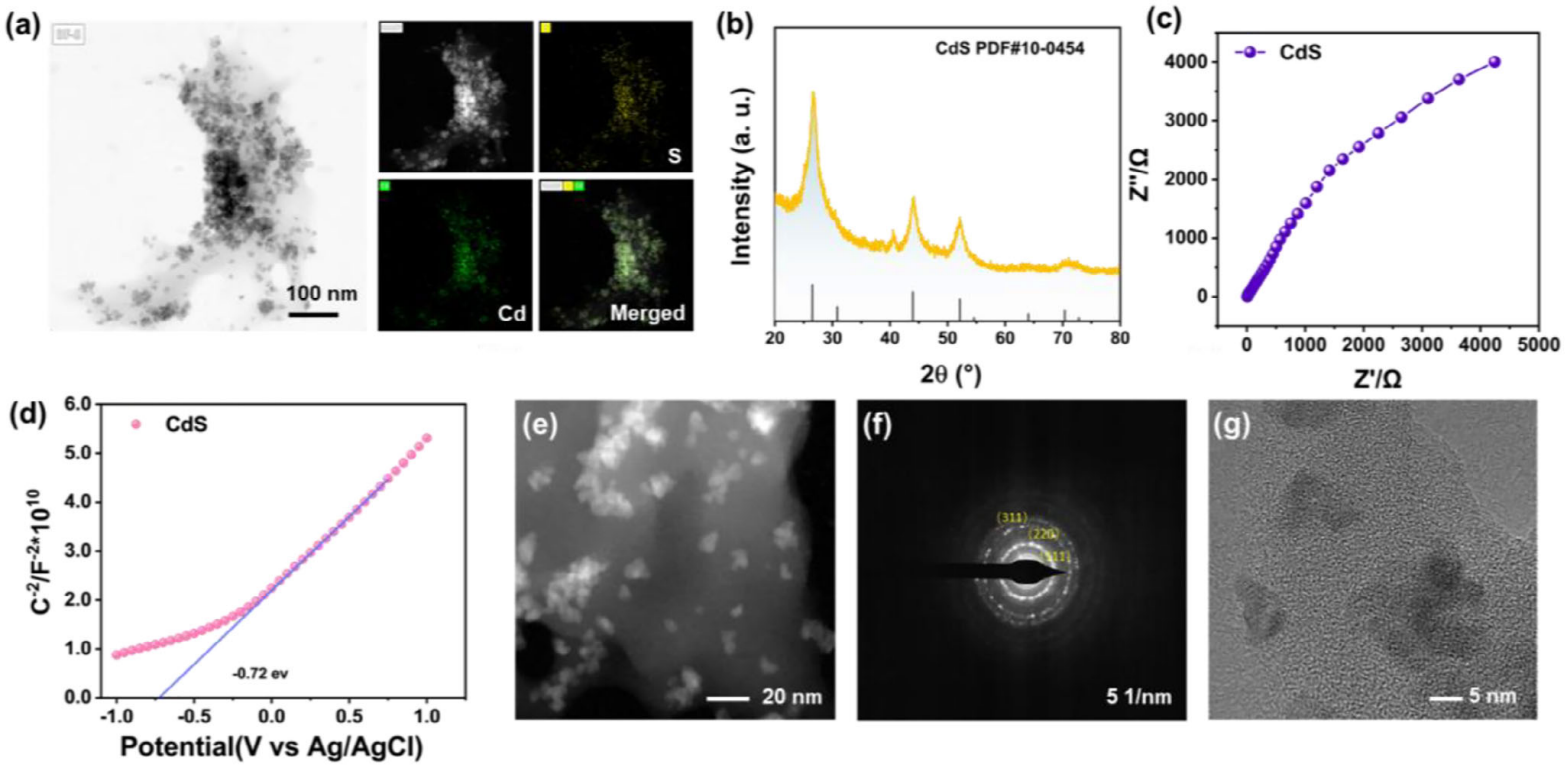
Structural and compositional characterization of CdS nanoparticles isolated from *B. subtilis*@CdS biohybrids. (a) Bright‐field transmission electron microscopy (BF‐TEM) image, high‐angle annular dark‐field scanning transmission electron microscopy (HAADF‐STEM) image, and TEM energy‐dispersive X‐ray spectroscopy (EDS) elemental mapping images of isolated CdS nanoparticles. (b) X‐ray diffraction (XRD) pattern of the isolated CdS nanoparticles, confirming their crystalline phase. (c) Electrochemical impedance spectroscopy yquist plot of the CdS (d) Mott‐Schottky plot of the composite, confirming the *n*‐type semiconducting behavior of the CdS. (e) Local HAADF‐STEM image revealing the nanoscale morphology of individual CdS particles. (f) Selected‐area electron diffraction (SAED) pattern, further verifying the crystalline nature of the CdS nanostructures. (g) High‐resolution TEM (HRTEM) image with the corresponding fast Fourier transform (FFT) pattern (inset), displaying clear lattice fringes characteristic of crystalline CdS.

### 2.2 Solar‐to‐Chemical Production by Biohybrids

#### 2.2.1 Solar‐Driven 2’‐FL Production Enhancement

Following successful construction of the semiconductor biohybrids, we next validated their capability to develop solar‐to‐chemical conversion. Specifically, *B. subtilis@*CdS was evaluated for synthesis of 2’‐FL. The engineered 2’‐FL‐producing strain synthesizes 2’‐FL from mannose‐6‐phosphate by a five‐enzyme cascade pathway that relies on reductant supply (Figure 5a) [44]. Here, GDP‐L‐fucose is the key precursor for 2’‐FL synthesis, and its production process is highly dependent on the reduction of nicotinamide adenine dinucleotide phosphate (NADPH), with particularly strong reducing power demand at the WcaG‐catalyzed step. As a result, this synthesis route is essentially a metabolic pathway that is highly sensitive to the level of cellular reducing power [45, 46], providing a direct metabolic interface through which photogenerated electrons can be harnessed to replenish reducing equivalents and enhance 2’‐FL production efficiency [47, 48, 49]. Based on the photoexcitation‐electron generation properties of CdS semiconductors. Upon light irradiation, the surface‐associated CdS semiconductors generate photoexcited electrons, which are transferred into the intracellular redox network and coupled to cellular metabolism, particularly driving the reduction of NADP^+^ to NADPH. The regenerated NADPH subsequently feeds into GDP‐L‐fucose biosynthesis, enabling the conversion of lactose to 2’‐FL through the WcaG‐dependent pathway (Figure 5a).

**FIGURE 5:**
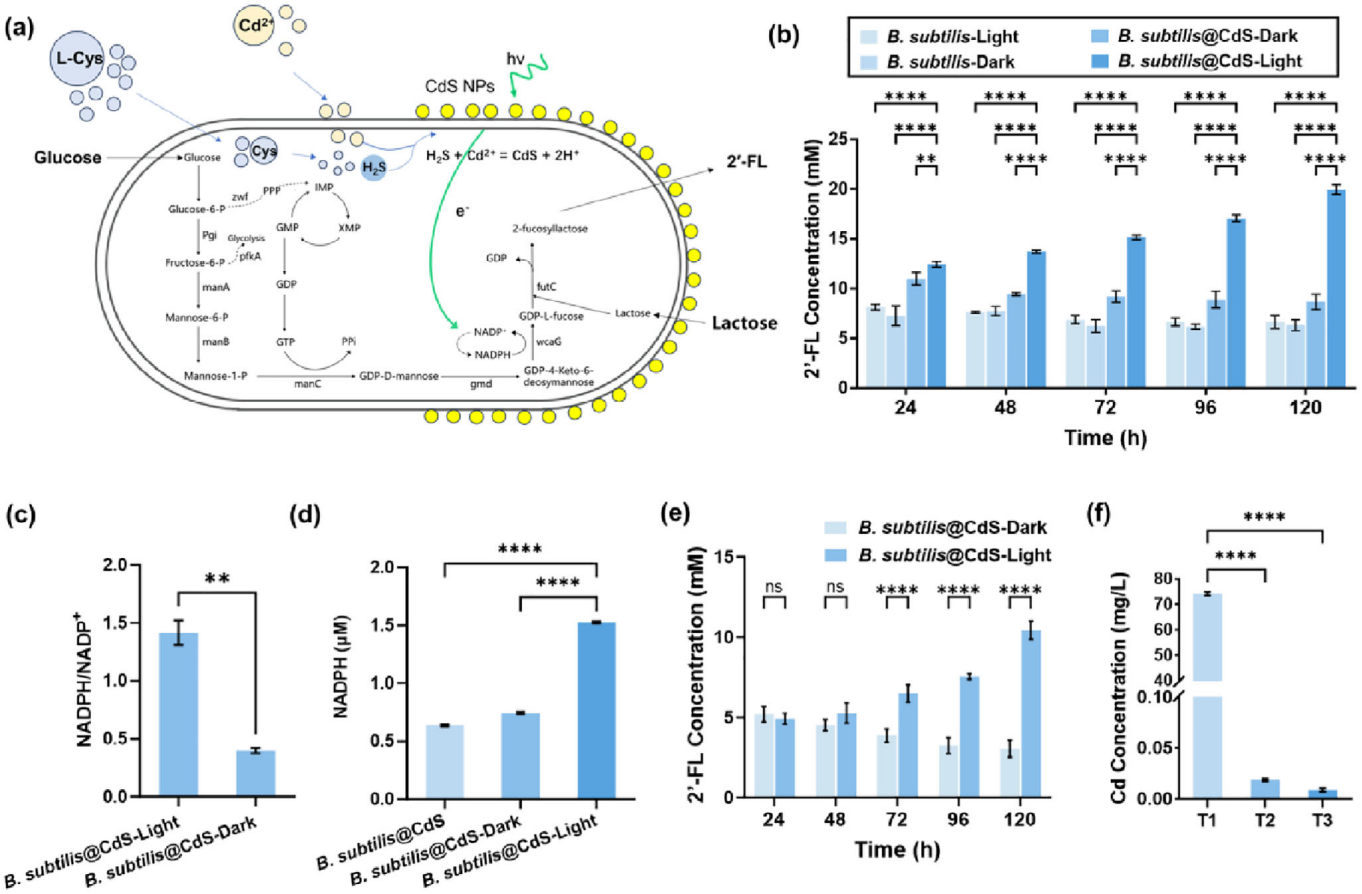
Light‐driven enhancement of 2’‐FL biosynthesis by *B. subtilis*@CdS semibiohybrids. *n* = 3 biological replicates. ^**^ *p* < 0.01, ^***^ *p* < 0.001, ^****^ *p* < 0.0001; The bars represent the mean±S.D. (a) Schematic illustration of semibiohybrid construction via in situ CdS self‐assembly on the bacterial surface and the corresponding mechanism for light‐driven enhancement of chemical production. (b) Time‐course profiles of 2’‐FL production by *B. subtilis* and *B. subtilis*@CdS hybrids under dark and illuminated conditions, showing a pronounced light‐dependent increase in product titer in the semi‐biohybrid system (Two‐way ANOVA). (c) Intracellular NADPH/NADP^+^ ratios after 48 h fermentation (Welch *t*‐test). (d) Evaluation of NADPH content in three systems (One‐way ANOVA). (e) Effects of exogenously added CdS nanoparticles on microbial production of target 2’‐FL (Two‐way ANOVA). (f) Multi‐step downstream purification strategy for Cd^2^^+^ removal from fermentation broth and ICP‐MS quantification of residual cadmium (One‐way ANOVA). T1: Fermentation broth supernatant (after centrifugation); T2: Na_2_S precipitation combined with diatomaceous earth adsorption; T3 Lewatit TP207 chelating resin ion exchange.

To examine whether this proposed light‐driven coupling translates into enhanced metabolic output, illumination experiments were performed in media supplemented with L‐Cys and Cd^2+^ to evaluate photo‐catalytically enhanced HMO production under 410 nm irradiation (Figure 5a, Figures S8 and S9). The 2’‐FL concentration in the *B. subtilis*@CdS‐Light group increased steadily with illumination time, reaching approximately 20 mmol L^−1^ at 120 h, which is nearly threefold higher than that of the *B. subtilis*‐dark group (Figure 5b and Figures S10 and S11, Table S2). To assess whether enhanced production was accompanied by changes in cellular activity, live/dead staining assays were conducted after prolonged illumination. The results showed that the viability of *B. subtilis*@CdS after 120 h of light exposure was higher than that of the *B. subtilis*‐dark group, indicating that CdS formation and continuous illumination did not impose substantial growth inhibition (Figure S12). The decline in 2’‐FL during fermentation stems from impaired biosynthesis and product degradation under carbon starvation. Initially, glycolysis and the tricarboxylic acid (TCA) cycle function normally, with NADPH supply exceeding biosynthetic demand, enabling continuous 2’‐FL accumulation. As the carbon source depletes, reduced glycolytic and TCA cycle flux limits NADPH regeneration below the level required for 2’‐FL synthesis, halting production. Concurrently, carbon starvation activates glycosidases that hydrolyze 2’‐FL into glucose and fucose, which re‐enter central metabolism for cell survival, further reducing 2’‐FL levels. In contrast, the *B. subtilis*@CdS light‐exposed group sustains NADPH generation via photocatalysis, maintaining 2’‐FL synthesis even under carbon limitation and achieving higher titers.

To investigate whether photoexcited electrons generated by the biogenic CdS nanoparticles can be harnessed for cellular energy metabolism, we measured the intracellular NADPH levels, NADPH/NADP^+^ ratios, and NADH/NAD^+^ ratios across different groups after 48 h of illumination (Figure 5c,d, and Figure S13). Both the NADPH level and the NADPH/NADP^+^ ratio in the illuminated *B. subtilis*@CdS group were significantly higher than those in the dark control, indicating that photoexcited electrons generated by CdS can be taken up by the cells and converted into cellular reducing power. Having established that illumination boosts intracellular reducing equivalents, we next investigated whether photogenerated electrons enter the cellular redox network through the respiratory chain. To this end, rotenone, a specific inhibitor of NADH dehydrogenase, was added during light‐driven fermentation. Upon rotenone treatment, 2’‐FL production, the NADPH/NADP^+^ ratio, and the NADH/NAD^+^ ratio all decreased significantly compared to the illuminated control without the inhibitor (Figure S14). These results suggest that the respiratory chain is related to the delivery of photogenerated electrons into the cellular redox network and contributes to light‐associated cofactor regeneration and the corresponding biosynthetic enhancement.

To validate the superiority of in situ CdS biosynthesis, we performed a control experiment using exogenously added commercialized CdS nanoparticles under identical photocatalytic fermentation conditions (Figure 5e). The resulting 2’‐FL titer was markedly lower than that of the *B. subtilis*@CdS system. This is because exogenous CdS particles tend to aggregate in the fermentation broth and lack direct electronic coupling with cellular metabolism, leading to inefficient photogenerated electron transfer for intracellular NADPH regeneration. In contrast, metabolically synthesized CdS is closely associated with the bacterial surface, enabling stable bio‐interface‐mediated energy transduction that is directly coupled to biosynthetic pathways, thereby achieving significantly enhanced 2’‐FL production. To address concerns regarding heavy metal residue risks of 2’‐FL, we assessed the distribution of cadmium throughout the fermentation system and subsequent purification steps the cadmium concentration in the fermentation broth was 74.11 mg/L; after purification, it was reduced to 8.52 µg/L in the isolated product, corresponding to a removal efficiency of 99.99% (Figure 5f).

#### 2.2.2 Solar‐Driven Surfactin Production Enhancement

Having validated solar‐driven 2’‐FL enhancement, we investigated this system's applicability to other *B. subtilis* engineered strains. Surfactin is a lipopeptide biosurfactant produced by nonribosomal peptide synthetases (NRPS) in *Bacillus* species. It exhibits exceptional surface‑active and emulsifying properties, leading to diverse applications across multiple sectors including biopharmaceutical, environmental remediation, enhanced oil recovery, cosmetics, and household products. The biosynthesis primarily demands substantial NADPH cofactors (Figure 6c). We successfully generated surfactin‐semibiohybrids, achieving photocatalyzed yield amplification. Bio‐SEM revealed dense nanoparticles predominantly packing on the surface of surfactin‐producing *B. subtilis* (Figure 6a). SEM‐EDS elemental mapping further confirmed uniform distribution of Cd and S across cellular surfaces (Figure 6b). Under illumination, the *B.subtilis*@CdS‐ light displayed a sustained and time‐dependent increase in surfactin accumulation. After 120 h of illumination, surfactin production reached 0.3750 g L^−1^, corresponding to a 1.66‐fold increase compared to dark controls (*B. subtilis*‐dark) (Figure 6d and Figure S15, Table S3). These results indicate that the semi‐artificial photosynthetic platform not only enhances production in 2’‐FL engineered strains but also improves surfactin biosynthesis in *B. subtilis*, highlighting its versatility for accelerating chemical biosynthesis across distinct metabolic products. For surfactin production, the cadmium concentration in the fermentation broth was 0.67 mg/L, and after purification, it was reduced to 8.54 µg/L in the isolated product, corresponding to a removal efficiency of 98.73% (Figure S16).

**FIGURE 6:**
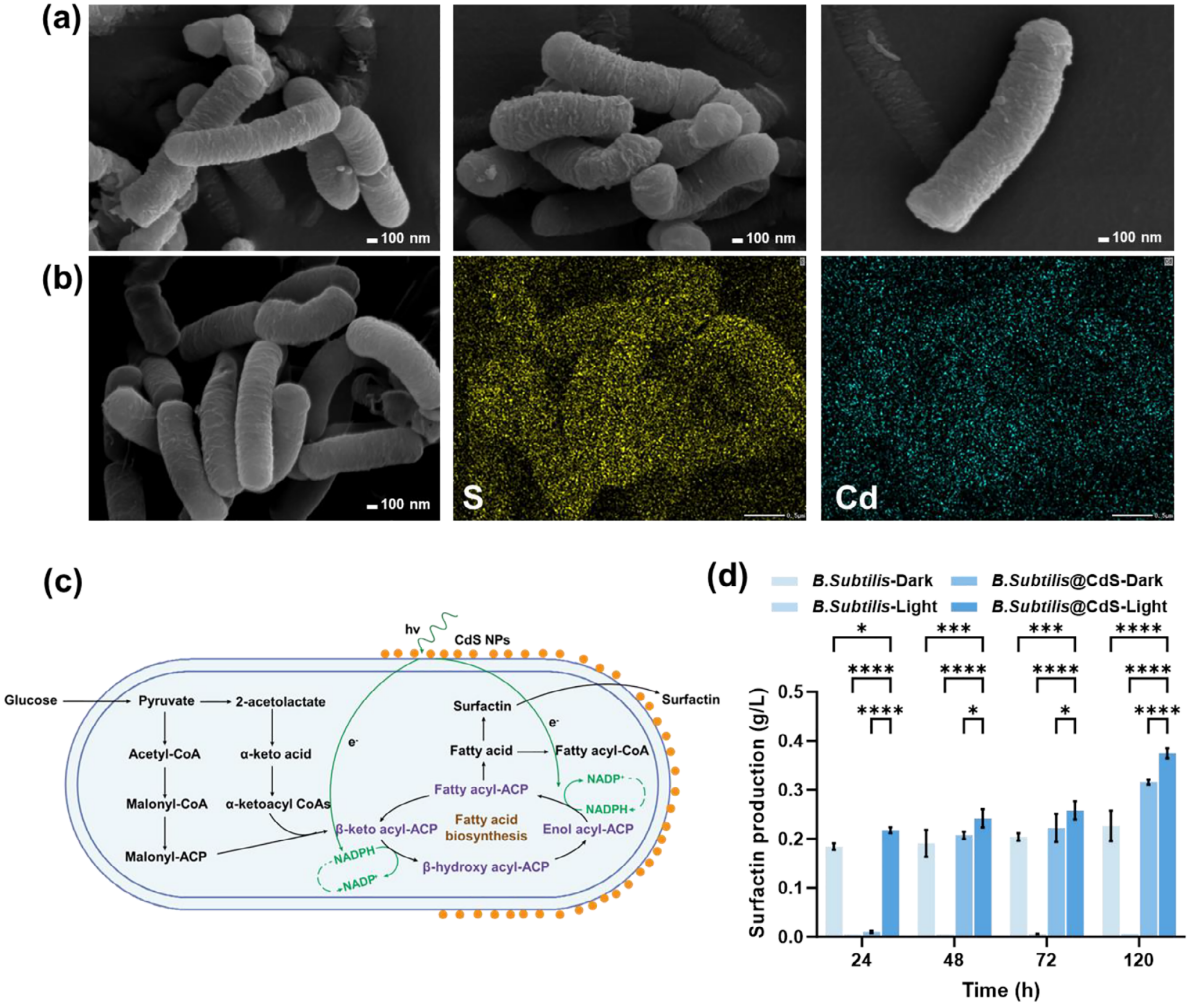
Characterization of *B. subtilis*@CdS semibiohybrids for light‐enhanced surfactin biosynthesis. (a) Scanning electron microscopy (SEM) image of *B. subtilis*@CdS semibiohybrids. (b) SEM‐energy‐dispersive x‐ray spectroscopy (SEM‐EDS) elemental mapping of the semibiohybrids. (c) Schematic illustration of the light‐enhanced surfactin biosynthesis pathway in *B. subtilis*@CdS semibiohybrids. (d) Time‐course profiles of surfactin production by *B. subtilis* and *B. subtilis*@CdS under dark and illuminated conditions. *n* = 3 biological replicates; Two‐way ANOVA. ^*^ *p* < 0.05, ^***^ *p* < 0.001, ^****^ *p* < 0.0001; The bars represent the mean±S.D.

### 2.3 Photoelectric Conversion and Metabolic Remodeling

The outstanding performance of the hybrid system is primarily attributed to the ability of CdS to absorb light and generate excited electrons, which are subsequently converted into cytosolic reducing equivalents and proton motive force. This leads to systemic alterations in the bacterial electron transport network and energy metabolic pathways, accompanied by enhanced extracellular electron transfer (Figure 7a). To further elucidate the involvement of photogenerated electrons in cellular metabolism, we performed non‐ targeted metabolomic profiling to examine metabolite changes in key biosynthetic pathways. Metabolomic profiling distinguished biohybrids from wild‐type *B. subtilis* by principal‐component analysis (PCA) (see heatmap in Figure S17 ). Differential metabolites were enriched in fatty‐acid, amino‐acid, sulfur, and energy metabolism, secretion, and redox processes based on the Kyoto Encyclopedia of Genes and Genomes (KEGG) and the GO analysis which reinforced the role of CdS in modulating electron transfer, energy metabolism, and transport processes (Figure 7 and Figures S18‐S22).

**FIGURE 7:**
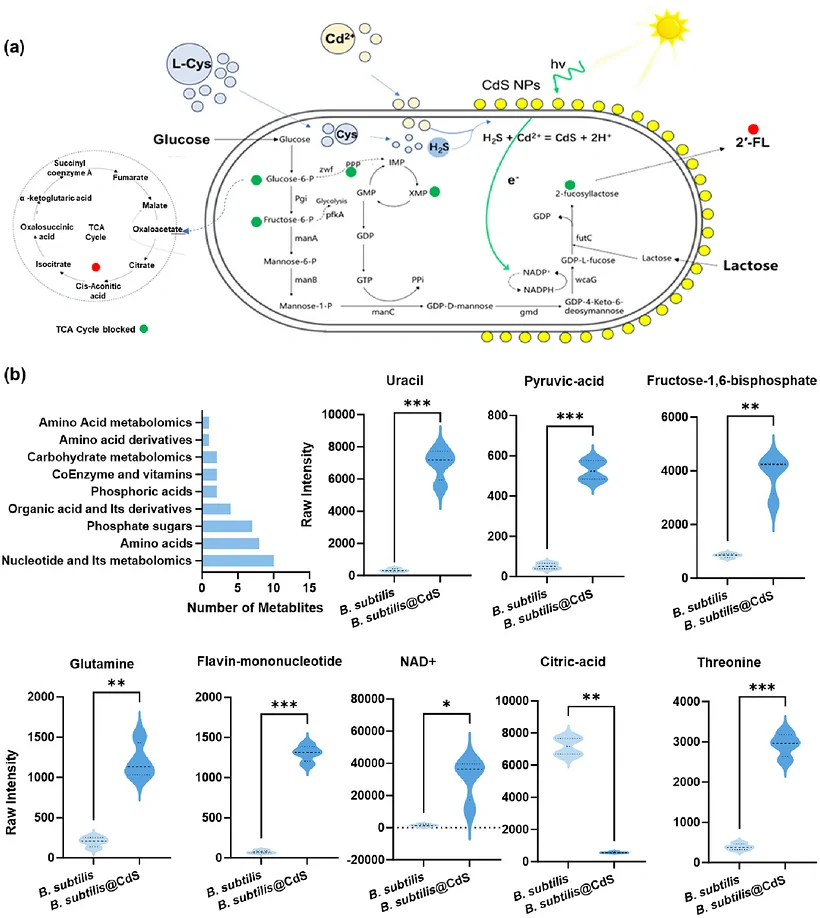
Light‐driven metabolic remodeling in semiconductor biohybrids. (a) Schematic illustration of the sunlight‐enhanced 2’‐FL biosynthetic pathway in biohybrids, highlighting photogenerated electron transfer from surface‐assembled CdS nanoparticles to intracellular redox metabolism, thereby promoting NADPH regeneration and precursor supply. (b) The abundance distribution comparison of 8 key metabolites between *B. subtilis* with *B. subtilis*@CdS. *n* = 4 biological replicates; Welch *t*‐test. ^*^ *p* < 0.05, ^**^ *p* < 0.01, ^***^ *p* < 0.001; The bars represent the mean±S.D.

Furthermore, targeted metabolomics mainly focuses on changes related to energy metabolism, based on multivariate VIP values (VIP > 1), univariate significance testing (*p* < 0.05), and FDR values in targeted metabolomics analysis, 37 significant differential metabolites related to energy metabolism were identified. 36 of these were upregulated in the *B. subtilis*@CdS group, while citrate, a core TCA cycle intermediate, was downregulated. These metabolites were primarily classified into nucleotides and their metabolites, amino acids and derivatives, and sugar phosphates, along with organic acid derivatives, coenzymes and vitamins, and carbohydrate metabolites. Key molecules in nucleotide metabolism, including uracil, inosine, guanosine diphosphatemannose (GDP), NAD^+^, and flavin mononucleotide (FMN), were significantly upregulated, with FMN and NAD^+^ showing the most pronounced increases, indicating enhanced intracellular energy transfer and redox turnover. Intermediates of the pentose phosphate pathway (PPP) accumulated significantly, providing pentose carbon skeletons for nucleotide and amino acid biosynthesis. Multiple amino acids, aspartate, lysine, and arginine, increased markedly, confirming activation of nitrogen metabolism and protein synthesis. In contrast, citrate was nearly depleted in the *B. subtilis*@CdS group, and pyruvate, lactate, and malate also showed significant differences between *B. subtilis with B. subtilis*@CdS. Collectively, coupling CdS with *B. subtilis* affects carbon, nitrogen, and nucleotide metabolism, these metabolomic changes suggest that light and CdS are associated with a shift in metabolic states related to energy metabolism and biosynthetic activity (Figure 7b).

### 2.4 Enhanced Biosynthesis via PbS and HgS Biohybrids

To assess the versatility of using endogenous H_2_S metabolism to construct surface‐associated semiconductor biohybrids, we further expanded the semi‐artificial biohybrids with different metal ions. *B. subtilis* successfully synthesized biohybrids not only with Cd^2+^ but also with Pb^2+^ and Hg^2+^, indicating that its H_2_S‐mediated biomineralization process has broader applicability. Photoelectrochemical analysis further demonstrated that both biohybrid systems exhibited strong photoactivity. The *B. subtilis*@PbS biohybrid generated a stable photocurrent density of approximately 0.13 µA cm^−2^ under intermittent illumination (Figure 8a), directly demonstrating the capability of cell‐surface‐assembled lead sulfide (PbS) to harvest light energy and generate charge carriers. Consistent with these observations, the *B. subtilis*@HgS biohybrid displayed even more pronounced photoactivity, yielding a photocurrent density of approximately 0.19 µA cm^−2^ under illumination (Figure 8b), indicating that the mercury sulfide (HgS) nanostructures assembled on the bacterial surface efficiently produce photogenerated electrons.

**FIGURE 8:**
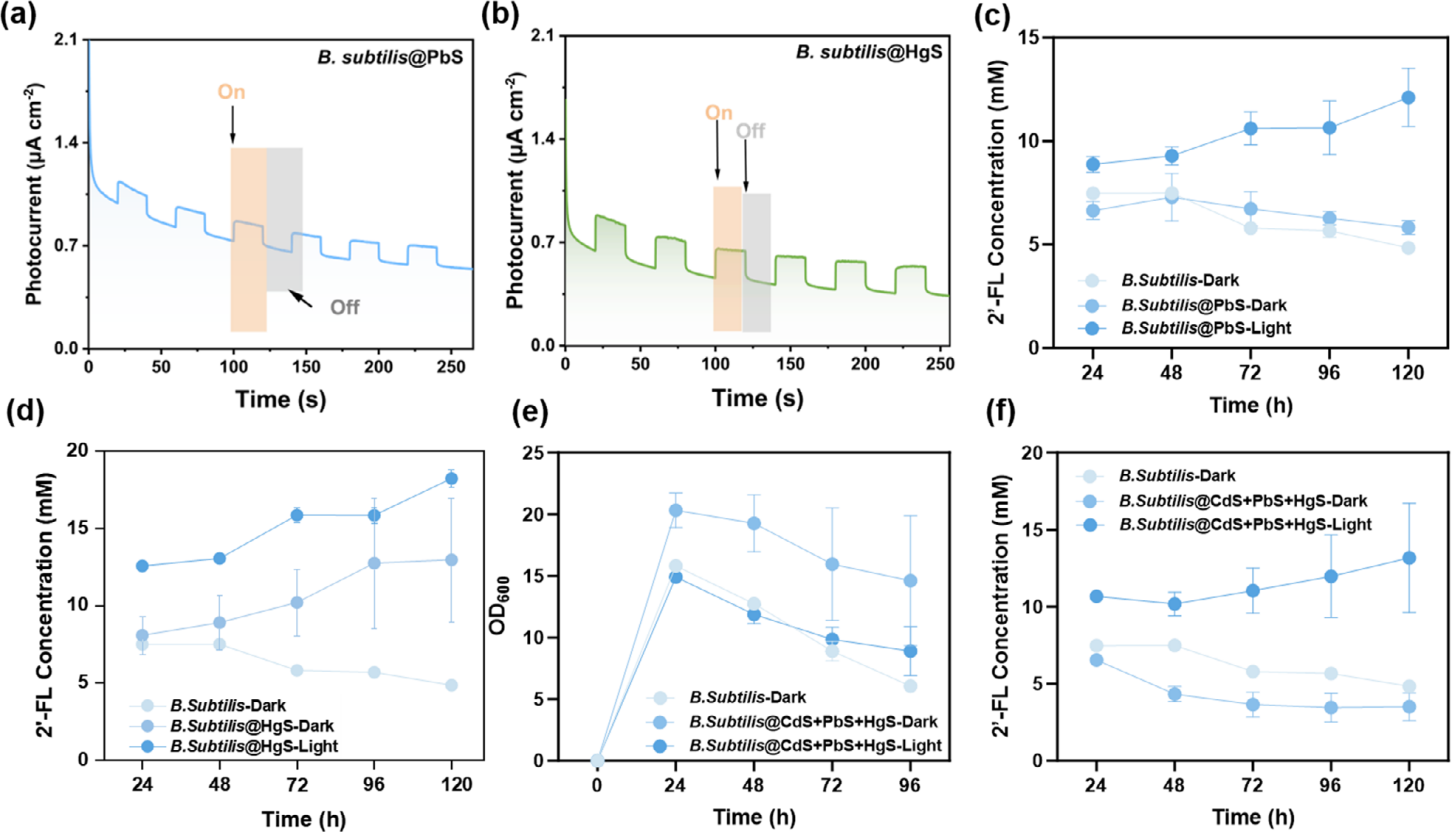
Extension of the CdS‐mediated biohybrid strategy to PbS, HgS, and multimetal sulfide systems. Representative photocurrent responses of (a) *B. subtilis*@PbS and (b) *B. subtilis*@HgS biohybrids under repeated light on/off cycles (20 s intervals, 100 mW cm^−2^). Production of 2’‐FL by (c) *B. subtilis*@PbS, and (d) *B. subtilis*@HgS biohybrids under dark and light conditions. (e) OD curve during mixed fermentation of *B. subtilis*@Cds‐HgS‐PbS. (f) Production of 2’‐FL by *B. subtilis*@Cds‐HgS‐PbS. *n* = 3 biological replicates, the bars represent the mean±S.D.

For the *B. subtilis*@PbS biohybrid, illumination markedly enhanced 2’‐FL biosynthesis. The 2’‐FL concentration in the illuminated *B. subtilis*@PbS‐Light group showed a 2.5‐fold increase relative to *B. subtilis* (Figure 8c). This titer was substantially higher than those of both the dark‐incubated *B. subtilis*@PbS‐Dark group and the *B. subtilis*‐Dark control, confirming that the stimulatory effect is strictly light‐dependent rather than attributable to PbS formation alone. Similarly, the illuminated *B. subtilis*@HgS culture exhibited a pronounced and sustained increase in 2’‐FL production over time (Figure 8d), with titers reaching nearly 3‐fold that of the corresponding dark‐grown control after 120 h of illumination. To further examine whether individual cells could simultaneously utilize multiple metal ions for semiconductor formation, *B. subtilis* was cultured in the concurrent presence of Cd^2+^, Pb^2+^, and Hg^2+^. The resulting *B. subtilis*@CdS‐PbS‐HgS biohybrid maintained stable cell growth under illumination (Figure 8e) while achieving a ∼3‐fold increase in 2’‐FL production relative to the corresponding dark‐grown control (Figure 8f, Table S4). These results demonstrate that a single *B. subtilis* cell can concurrently exploit multiple metal ions via the H_2_S pathway to assemble multicomponent sulfide semiconductors on the cell surface. In addition, given that cysteine metabolism is broadly conserved across microorganisms, this in situ biomineralization strategy is readily generalizable to other hosts, such as constructing *E. coli*@CdS biohybrids, expanding the versatility of the non‐invasive assembly platform (Figure S23).

## 3 Discussion

This study demonstrates an efficient strategy for enhancing the production of high‐value compounds in *B. subtilis* through the in situ construction of light‐driven semiconductor biohybrids [48, 50]. By harnessing the endogenous H_2_S metabolic pathway, metal ions are rapidly converted into metal sulfide semiconductors on the cell surface, enabling the formation of a well‐integrated bio‐semiconductor interface and efficient utilization of photogenerated electrons [50]. Unlike conventional biohybrid fabrication approaches that rely on physical mixing or ex situ nanoparticle deposition, this in situ self‐assembly strategy ensures uniform semiconductor coverage and stable cell‐material contact, thereby overcoming common limitations such as nanoparticle aggregation, low cellular adsorption efficiency, and heterogeneous interfacial electron transfer [48]. Our results demonstrate that cell‐surface‐assembled CdS effectively injects photogenerated electrons into cellular metabolism [45, 51], leading to markedly enhanced biosynthetic outputs. This conclusion is supported by multiple lines of evidence: (i) *B. subtilis*@CdS exhibits pronounced photocurrent generation under illumination; (ii) light exposure results in substantial increases in both 2’‐fucosyllactose (2’‐FL) and surfactin production; and (iii) the enhanced production was achieved without a proportional increase in biomass, demonstrating that photogenerated electrons enhance single‐cell metabolic efficiency rather than promoting growth. Collectively, these observations indicate that the primary role of the semiconductor biohybrid is metabolic modulation rather than growth promotion. This behavior is consistent with a mechanism in which photogenerated electrons enhance intracellular NADPH availability, thereby promoting biosynthesis.

Compared with conventional genetic engineering strategies, this materials‐driven approach offers a complementary and potentially faster route to improving microbial production. Conventional metabolic engineering for pathway optimization is a time‐consuming process, often requiring 6 to 18 months for promoter screening, RBS tuning, and circuit balancing to alleviate metabolic burden [45]. For example, a recent study aimed at improving surfactin production in *B. subtilis* employed data‐driven active learning combined with multiple “design‐build‐test‐learn” cycles to optimize culture conditions, achieving only a 1.6‐fold increase after extensive iteration. Similarly, in engineered 2’‐FL production systems, the introduction and optimization of heterologous glucose transporters to enhance precursor supply resulted in a final yield improvement of approximately 1.9‐fold [52]. These examples illustrate that even moderate gains (1.6 ∼ 2‐fold) through traditional genetic approaches often require substantial time and engineering complexity. In contrast, our non‐genetic approach achieves pronounced productivity enhancement through fermentation‐level optimization, particularly by regulating L‐cysteine and metal ion concentrations to control semiconductor formation. Notably, a 3‐fold increase in 2’‐FL production and a 1.66‐fold enhancement in surfactin yield were achieved without any genetic modification, substantially shortening the development cycle and highlighting the potential of semiconductor‐mediated metabolic regulation as an alternative route to strain engineering.

Our findings further reveal the effectiveness of this strategy across distinct metabolic pathways. Both 2’‐FL biosynthesis, a GDP‐fucose‐ and NADPH‐dependent process, and surfactin production, an NADPH‐intensive nonribosomal peptide pathway, were significantly enhanced. Moreover, *B. subtilis* was capable of simultaneously assembling multicomponent sulfide semiconductors incorporating Cd^2+^, Pb^2+^, and Hg^2+^, underscoring the remarkable flexibility of this H_2_S‐driven biomineralization pathway. This finding suggests the feasibility of constructing compositionally tunable semiconductor interfaces on living cells, opening new avenues for tailoring light absorption and electron transfer properties to meet specific metabolic demands [50]. The core principle of using photoelectrons to enhance reducing power and energy supply is applicable to a wide range of NADPH‐dependent bioprocesses. This approach thereby provides a versatile strategy for the production of various high‐value compounds and other secondary metabolites, without the need for extensive genetic manipulation [48].

However, several limitations warrant further investigation. First, while heavy metal handling is manageable under laboratory conditions, the long‐term stability and metal accumulation in the biohybrid system remain critical concerns for industrial deployment [45]. Future studies could explore microbial remediation strategies and leverage metal‐rich effluents as a resource for biohybrid synthesis, integrating wastewater remediation with the production of value‐added chemicals. Second, the current platform has been validated for a limited set of products; future work should evaluate its applicability to broader NADPH/NADH‐intensive biosynthetic pathways, such as terpenoids and pharmaceutical intermediates. Third, although this platform reduces reliance on genetic modification, its integration with targeted metabolic engineering, combining enhanced cofactor regeneration with optimized metabolic fluxes, may yield synergistic benefits and merits further exploration.

In conclusion, this study developed an efficient, non‐genetic strategy to enhance microbial biosynthesis through the in situ self‐assembly of semiconductor biohybrids mediated by endogenous microbial metabolism. By leveraging the H_2_S metabolic pathway, *B. subtilis* gains the ability to convert light energy into metabolic reducing power, thereby substantially improving the production efficiency of value‐added compounds. The successful extension of this approach to multiple metal sulfides and *E.coli* highlights its great potential, positioning semiconductor biohybrids as a platform for biomanufacturing and waste valorization, offering an alternative to traditional metabolic engineering for boosting the production of value‐added chemicals.

## 4 Materials and Methods

### 4.1 Materials and Reagents

Cadmium chloride anhydrous (CdCl_2_), lead (II) acetate trihydrate (Pb(CH_3_CO_2_)_2_·3H_2_O), mercury (II) nitrate (Hg(NO_3_)_2_), and L‐cysteine (L‐Cys) were purchased from Macklin Biochemical Co., Ltd. (Shanghai, China). The 2’‐fucosyllactose (2’‐FL) standard was obtained from Biosynth (Shanghai, China). The surfactin standard was sourced from Yuanye Bio‐Technology Co., Ltd. (Shanghai, China). All other chemicals, unless otherwise specified, were purchased from Sinopharm Chemical Reagent Co., Ltd. (China).

### 4.2 Strain and Culture Conditions

The strains used in this study were two engineered *Bacillus subtilis*: *Bacillus subtili*s‐2’‐FL (for 2’‐FL production) and *Bacillus subtilis*‐Surf (for surfactin production). *Bacillus subtilis* activated in LB medium to the logarithmic phase were inoculated into fresh sugar‐containing TB medium for scale‐up cultivation, followed by incubation at 37°C with shaking at 220 rpm. The wavelength of the light was 310, 370, and 410 nm, and the light intensity levels of 75 W/m^2^, and irradiance was measured directly at the sample position (liquid surface level) using a calibrated radiometer. Fermentation vessels were 250 mL conical flasks. Flasks were sealed with breathable membranes to ensure gas exchange while preventing contamination. Temperature was maintained at 37°C using a temperature‐controlled incubator shaker (220 rpm), with temperature fluctuations during irradiation kept within ±1.0°C. The initial pH was adjusted to 6.25 and monitored throughout the fermentation process. All fermentation experiments (2’‐FL and surfactin) were performed as three independent biological replicates. Unless otherwise stated, data are presented as mean ± SD of the three independent fermentations (*n* = 3 biological replicates).

### 4.3 Construction of Biohybrid Systems and Determination of H_2_S Content

Sterile aqueous solutions of L‐cysteine (L‐Cys) and metal ions (Cd, Pb, Hg) were prepared separately. *B. subtilis* cultivation was initiated as described in section strain and culture conditions. When the scaled‐up *B. subtilis* cultures in TB medium reached OD600 = 10, isopropyl β‐D‐1‐thiogalactopyranoside (IPTG) was added to a final concentration of 0.2 mM to initiate induction. Simultaneously with induction, pre‐prepared L‐Cys solution was introduced. Following 2 h of incubation, metal ion solutions were supplemented. The cultures were maintained at 37°C with 220 rpm shaking for 5 days to form semiconductor‐biohybrid materials. Samples were collected at 24 h intervals and stored at −20°C for subsequent analysis. H_2_S gas was quantified using an IFU hydrogen sulfide Dräger tube (Dräger) and a pump‐suction gas detector.

### 4.4 NAD^+^/NADH and NADP^+^/NADPH Concentrations and Ratios Assay

Intracellular NAD^+^/NADH and NADP^+^/NADPH concentrations and ratios were measured at 48 h using the NAD^+^/NADH Assay Kit and the NADP^+^/NADPH Assay Kit (Beyotime Biotechnology, Shanghai, China), respectively. Samples were collected and immediately processed for extraction with the provided extraction buffer according to the manufacturer's instructions. All extraction steps were performed under dark conditions to minimize degradation. Each condition was assessed with three biological replicates (*n* = 3), each measured in biological technical duplicate.

### 4.5 Construction of *Bacillus Subtilis* Mutants

The plasmid and strains constructed and used in this study are listed in Table 1. And the primers used in this study are listed in Table S1 [53].

**TABLE 1:** Plasmid and strains used in the study.

Plasmid or Strain	Description	Reference
Plasmid		
p7Z6	A pMD18‐T derived plasmid, containing the *lox71‐zeo‐lox66 cassette*.	[54]
pDG148	Amp^r^, Kan^r^; an *E. coli–B. subtilis* temperature‐sensitive shuttle plasmid containing the P*spac*‐*cre* fragment.	[54]
Strain		
*Bacillus subtilis* 168	The parental strain	Our Lab
BS‐KO1	*Bacillus subtilis* 168 Δ*mccB*	This study
BS‐KO2	*Bacillus subtilis* 168 Δ*mccB*,Δ*metC*	This study
BS‐KO3	*Bacillus subtilis* 168 Δ*mccB*,Δ*metC*,Δ*cysK*	This study

### 4.6 Analytical and Characterization Methods

#### 4.6.1 SEM‐EDS

Fresh specimens were fixed overnight at 4°C in 2.5% glutaraldehyde solution. The fixative was removed by aspiration, followed by three 15‐min washes with 0.1 m phosphate‐buffered saline (PBS, pH 7.0). Post‐fixation was performed with 1% osmium tetroxide for 1–2 h in a fume hood. After careful removal of the osmium solution, samples were rinsed thrice with PBS (15 min per wash). Dehydration was carried out through a graded ethanol series (30%, 50%, 70%, 80%, 90%, and 95%; 15 min per concentration) followed by two 20‐min treatments with absolute ethanol. Samples were then transitioned in ethanol/isoamyl acetate (1:1, v/v) for 30 min and pure isoamyl acetate for 1 h or overnight. The samples were dried using a critical‐point dryer (Leica CPD300) and imaged by SEM (JEOL JSM‐IT800) with EDS after gold spraying.

#### 4.6.2 Nanoparticles Extraction

The collected precipitate was resuspended in 50 mM Tris‐HCl buffer (pH 7.5) and sonicated for 3 h using a probe ultrasonicator (Scientz‐IID) in an ice bath. Nanoparticles were then collected by centrifugation at 8,000 rpm for 1 h. The particles were dispersed in Li_2_S/formamide solution (5 g L^−1^) via 15‐min sonication, followed by addition of didodecyldimethylammonium bromide (DDAB) in toluene (50 g L^−1^). The mixture was stirred overnight at room temperature. DDAB‐functionalized nanoparticles were collected from the upper organic phase, washed three times with acetone and ethanol to remove excess surfactant, and centrifuged at 12 000 rpm for 10 min. The purified nanoparticles were lyophilized for subsequent analysis.

#### 4.6.3 TEM‐EDS

The collected sample powder was dispersed in ethanol for 30 min, deposited onto copper TEM grids, and air‐dried at ambient temperature. Following gold sputter‐coating (5 nm thickness), material morphology was examined using transmission electron microscopy (Talos F200X G2) operated at 200 kV.

#### 4.6.4 FT‐TR

To analyze changes in the functional groups of *Bacillus subtilis* after Cd^2+^ adsorption, the sample powder was thoroughly ground with KBr in an agate mortar. The homogeneous mixture was then transferred to a die and compressed using a hydraulic press to form translucent pellets. The pellets were analyzed by FTIR (Vertex 70, Bruker) over the wavenumbers range of 400–4000 cm^−1^ with a resolution of 4 cm^−^^1^.

#### 4.6.5 XRD and Photoelectrochemical Analysis

To determine the crystal structure of semiconductor nanoparticles, XRD patterns were detected using an XRD instrument (Areis, PANalytical) at 20°–80°. Photoelectrochemical analysis. Electrochemical analysis was performed using an electrochemical workstation (CHI760E, Shanghai Chenhua). The semiconductor biohybrid material was loaded onto indium tin oxide (ITO) to form a uniform 1 × 1.3 cm^2^ film by drop casting. Electrochemical impedance spectroscopy (EIS) was performed at a bias potential of −0.3 V (vs. Ag/AgCl) with a frequency range of 100 kHz–0.1 Hz and an AC amplitude of 10 mV. Mott–Schottky analysis was conducted by sweeping the potential from −1 to +1 V at a fixed frequency of 2000 Hz. All electrochemical measurements were carried out in 0.5 m Na_2_SO_4_ aqueous solution using an Ag/AgCl reference electrode. A 300 W Xe‐arc lamp (Newport) equipped with an infrared cut‐off filter was employed as the solar simulator. The light intensity was calibrated to 1 sun illumination (100 mW/cm^2^).″

#### 4.6.6 UV–Vis DRS

UV–vis DRS (UV‐3600i Plus, Japan Shimadzu) was used to measure the direct bandgap. Powdered samples in quartz cells (thickness ≥  2 mm) were analyzed against a BaSO_4_ reference over 200–800 nm (1 nm interval).

Conversion of reflectance data by the Kubelka–Munk function:

$$F\left(R\right)=\frac{{\left(1-R\right)}^{2}}{2R}$$

The bandgap energy of the calculated material was obtained by Tauc plot method.

#### 4.6.7 Non‐Targeted Metabolomics Analysis

For non‐targeted metabolomics analysis, eight samples (2 mL each, *n* = 4 biological replicates) of *B. subtilis*‐CdS biohybrids and wild‐type *B. subtilis* cultures at 72 h fermentation were collected. Samples were centrifuged at 8000 × g for 2 min. Cell pellets were preserved in liquid nitrogen. Metabolomic analysis was conducted by Bioyigene Co., Ltd. (Wuhan, China); Thawed samples on ice were thoroughly ground under liquid nitrogen (60 Hz, 30 s). Aliquots (40 mg) were transferred to centrifuge tubes, suspended in 400 µL of 80% aqueous methanol, and subjected to three freeze‐thaw cycles: snap‐freezing in liquid nitrogen (5 min), thawing on dry ice with vortex mixing, and ultrasonication (6 min). After centrifugation (4°C, 5,000 × g, 15 min), supernatants were lyophilized and reconstituted in 10% methanol. Finally, 2 µL of supernatant was pipetted and injected into a Thermo UHPLC‐Q Exactive HF‐X Vanquish Horizon system, equipped with a Hypesil Gold column C18 (100 × 2.1 mm, 1.9 µm; Thermo, USA), using a 12‐min linear gradient at a flow rate of 0.2 mL/min. The eluents for the positive polarity mode were eluent A (0.1% TFA in Water) and eluent B (Methanol). The eluents for the negative polarity mode were eluent A (5 mm ammonium acetate, pH 9.0) and eluent B (Methanol). The solvent gradient was set as follows: 2% B, 1.5 min; 2%–85% B, 3 min; 85%–100% B, 10 min; 100%–2% B, 10.1 min; 2% B, 12 min. Q ExactiveTM HF mass spectrometer was operated in positive/negative polarity mode with spray voltage of 3.5 kV, capillary temperature of 320°C, sheath gas flow rate of 35 psi, aux gas flow rate of 10L/min, S‐lens RF level of 60, Aux gas heater temperature of 350°C. After UHPLC‐MS/MS analyses, the raw data were imported into the Compound Discoverer 3.3 (CD3.3, ThermoFisher) for peak detection, alignment, and identification. Lipidomic features detected in at least 80% of any set of samples were retained. After filtering, minimum metabolite values were imputed for specific samples in which the metabolite levels fell below the lower limit of quantitation and each metabolic feature were normalized by sum. The response intensity of the sample mass spectrum peaks was normalized by the sum normalization method, and the normalized data matrix was obtained for subsequent analysis.

## Author Contributions

Conceptualization: A.T., J.Z., and X.Z. Methodology: A.T., J.Z., M.Q. and S. Y. Investigation: J.Z., A.T., W.L. and M. S. Visualization: J.Z., A.T., and Y. G., Supervision: X.Z. Writing – original draft: A.T., Writing – review & editing: J.Z., M.Q., and X.Z. Project administration: X.Z., S. H. Funding acquisition: X.Z.

## Conflicts of Interest

The authors declare no conflicts of interest.

## Supporting information




**Supporting File**: advs78265‐sup‐0001‐SuppMat.docx.

## Data Availability

The data that supports the findings of this study are available in the supplementary material of this article.
