# Supplementary material for: Semi‐Artificial Photosynthesis Enables Light‐Driven Biomanufacturing in Non‐Photosynthetic Bacteria
Source: Adv Sci (Weinh). 2026 Oct 14:e78265. Online ahead of print. doi: 10.1002/advs.78265 (PMC13669208; doi:10.1002/advs.78265)
Supplement: Supplementary file 1 [file ADVS-9999-e78265-s001.docx]

Supplementary Materials for

**Semi-artificial photosynthesis enables light-driven biomanufacturing in**

**non-photosynthetic bacteria**

Jie Zheng ^a,b,#^, Anqi Tong ^a,#^, Wenxin Li ^a,#^, Meng Qiao ^b^, Yang Gu ^a^, Muhammad Sohail ^a^, Shan He ^d^, Sheng Yang ^c^, Xing Zhang ^a,^*

^a^ State Key Laboratory of Microbial Technology, School of Food Science and Pharmaceutical Engineering, Jiangsu Key Laboratory for Peptide Biosynthesis， Nanjing Normal University, Nanjing 210023, China

^b^ Research Center of Pharmaceutical and Synthetic Biology/Department of Food Quality and Safety, College of Engineering, China Pharmaceutical University, Nanjing 211198, China

^c^ Key Laboratory of Synthetic Biology, CAS Center for Excellence in Molecular Plant Sciences, Chinese Academy of Sciences, Shanghai 200032, China

^d^ Faculty of Science and Technology, Charles Darwin University, Brinkin, NT 0810, Australia

^#^These authors contributed equally to this work.

^*^ To whom correspondence should be addressed.

Email: X.Z. ([zhangxing@njnu.edu.cn](mailto:zhangxing@njnu.edu.cn))

**Disruption H_2_S synthesis pathway in *Bacillus subtilis***

To verify the contribution of hydrogen sulfide derived from L-cysteine metabolism to the formation of cadmium sulfide in *Bacillus subtilis*, we disrupt its H_2_S synthesis pathway. It has been reported that *mccB*, *metC*, and *cysK* are related to the hydrogen sulfide metabolic pathway in *Bacillus subtilis* (Marie-Françoise et al., 2007). Therefore, we constructed a series of mutants by sequentially interrupting these three genes using the Cre-LoxP method. First, upstream and downstream homologous arms of approximately 500 bp were designed based on the target genes. These arms were amplified using the *B. subtilis* genome as a template. The LoxP-Zeocin-LoxP (LZL) fragment was amplified using the p7Z6 plasmid ^[1]^ as a template. The upstream and downstream homologous arms were then fused with the LZL fragment via overlap PCR to obtain the homologous recombination fragment. The fragment was introduced into *B. subtilis* competent cells. After recovery, the cells were plated on LB agar containing Zeocin (40 μg/mL) and incubated overnight at 37°C. After the verification of colony PCR amplification and Sanger sequencing of the target locus, positive clones were used to prepare competent cells to transform the pDG148 plasmid ^[2]^ expressing cyclization recombinase (Cre) for excising the antibiotic resistance marker. The transformants were plated on LB agar containing Kan (50 μg/mL) and incubated overnight at 37°C. Then pDG148 transformants were inoculated into liquid LB medium supplemented with 0.2 mmol·L^-1^ IPTG and 50 μg/mL kanamycin, and cultured at 30°C with 220 rpm for 24 hours. Then 1 μL culture was transferred into 2 mL fresh liquid LB medium and shaken at 50°C with 220 rpm for 10 hours to eliminate pDG148 plasmid. The cells were then streaked onto LB agar plates without antibiotics. Subsequently, individual colonies were sub-cultured onto three distinct types of LB agar plates: one containing zeocin, one containing kanamycin, and one without any antibiotic. Only colonies that grew exclusively on the antibiotic-free plate, but not on the zeocin- or kanamycin-containing plates, were identified as successful knockout mutants with concomitant loss of the pDG148 plasmid. These verified mutants were then used as the parental strains to repeat the aforementioned gene knockout procedure for the subsequent gene, continuing iteratively until all three target genes were disrupted.

**Cell Viability Analysis**

Bacterial cell viability was assessed using dual fluorescent staining with SYTO 9 and propidium iodide (PI). SYTO 9 is a membrane-permeant nucleic acid dye that stains all bacterial cells and emits green fluorescence, whereas PI is a membrane-impermeant dye that penetrates only cells with compromised membranes. Upon entering dead cells, PI competitively quenches SYTO 9 fluorescence and emits red fluorescence. Briefly, 1 mL of fermentation broth was collected and centrifuged at 12,000 × g for 5 min at 4°C. The supernatant was discarded, and the cell pellet was washed twice with sterile 0.85% saline solution and resuspended. Subsequently, 100 μL of the cell suspension was mixed with an equal volume of SYTO 9/PI staining solution prepared according to the manufacturer’s instructions (e.g., LIVE/DEAD® BacLight™ Bacterial Viability Kit). The mixture was incubated at room temperature for 15-20 min in the dark. After incubation, 5 μL of the stained cell suspension was placed on a microscope slide, covered with a coverslip, and immediately observed using a fluorescence microscope equipped with FITC (for SYTO 9, excitation/emission ≈ 488/520 nm) and TRITC (for PI, excitation/emission ≈ 535/617 nm) filter sets. Live cells exhibited green fluorescence, whereas dead cells displayed red fluorescence.

**NADPH/NADP^+^ assay**

The NADPH/NADP^+^ assay kit detects NADP^+^, NADPH, and their ratio in samples based on the following principles: This kit detects NADP^+^, NADPH, and their ratio in samples based on the following principles. First, glucose-6-phosphate (G6P) is oxidized to 6-phosphogluconate (6-PG) by glucose-6-phosphate dehydrogenase (G6PDH), during which NADP⁺ is reduced to NADPH. The resulting NADPH then reduces WST-8 to an orange-yellow formazan via the electron coupling reagent 1-mPMS (1-Methoxy-5-methylphenazinium methyl sulfate), with a maximum absorption peak at approximately 450 nm. To measure NADPH alone, the sample is heated in a 60°C water bath for 30 minutes to decompose NADP^+^ while preserving NADPH, after which the NADPH reduces WST-8 to formazan for colorimetric quantification. Finally, the amount of NADP^+^ is obtained by subtracting the NADPH value from the total NADP^+^/NADPH value, and the NADP^+^/NADPH ratio is calculated accordingly.

**NADH/NAD^+^ assay**

This kit detects NAD^+^, NADH, and their ratio in samples based on the following principles: First, ethanol is oxidized to acetaldehyde by alcohol dehydrogenase (ADH), during which NAD⁺ is reduced to NADH. The resulting NADH then reduces WST-8 to an orange-yellow formazan via the electron coupling reagent 1-mPMS, with a maximum absorption peak at approximately 450 nm. The amount of formazan produced is proportional to the total amount of NAD^+^/NADH in the sample. To measure NADH alone, the sample is heated in a 60°C water bath for 30 minutes to decompose NAD^+^ while preserving NADH, after which the NADH reduces WST-8 to formazan for colorimetric quantification. Finally, the amount of NAD^+^ is obtained by subtracting the NADH value from the total NAD^+^/NADH value, and the NAD^+^/NADH ratio is calculated accordingly.

**Table S1. Primers used in this study**

| Primer name | Sequence |
| --- | --- |
| LZL-F(T1) | GGTACCCGGGGATCCTCTAG |
| LZL-R(T2) | GCATGCCTGCAGGTCGAC |
| komccB-UP-F(T3) | CGTTTTGCAATTTAAAAACCGAGTAAATCC |
| komccB-UP-R(T4) | GGTAGAATCTCTAGAGGATCCCCGGGTACCATCAGATGCCTCCTTCGTATATTTGTTTG |
| komccB-DN-F(T5) | CGGTAGAATCGTCGACCTGCAGGCATGCCAAATGGATAGAATTACTTTCTCTGTAAGCC |
| komccB-DN-R(T6) | GCGTAATGGCTGGGACTGAAC |
| komccB-Id-F(T7) | ACAGCAAATTATGCAAGCGC |
| komccB-Id-R(T8) | GCCTGTCACCAACCTCATCA |
| kometC-UP-F(T17) | TAACGATCTGCTTGCTGGACTT |
| kometC-UP-R(T18) | GTAGAATCTCTAGAGGATCCCCGGGTACCATGAAACAGCTCCCTCTTTGACCTGACATA |
| kometC-DN-F(T35) | GTAGAATCGTCGACCTGCAGGCATGCTGAGTGTCGGCGTCGAACATGCAGACGATTTAG |
| kometC-DN-R(T36) | CCGAGTATCAGGAAAGACTTGAGAAAGATGAAGAATATCATTTTGGC |
| kometC-Id-F(T21) | GACAAACCCCCTCATGCAGG |
| kometC-Id-R(T22) | ATGCTGACATGAGAACGGAG |
| kocysK-UP-F(T39) | GCTGTCGCTGCCGAAAGATCTACAGACAG |
| kocysK-UP-R(T40) | GAACGGTAGAATCTCTAGAGGATCCCCGGGTACCCCGCACTGTTTTCATCTGCAAGGCG |
| kocysK-DN-F(T41) | CGGTAGAATCGTCGACCTGCAGGCATGCGCCATCATTCCAAGTAACGGCGAACGTTATC |
| kocysK-DN-R(T42) | GTCAGGGAACTCATGATTCGTTTCTGTTTCCAGTG |
| kocysK-Id-F(T33) | ATGAACATACGACGGAGAAAGAG |
| kocysK-Id-R(T34) | GGGAAGCTCAGTCAACCACA |

**Table S2.** Product parameter of 2'-FL in *B. subtilis* and *B. subtilis*@CdS

|  | *B. subtilis* | | *B. subtilis*@CdS | |
| --- | --- | --- | --- | --- |
|  | Light | Dark | Light | Dark |
| Fermentation time (h) | 120 | 120 | 120 | 120 |
| Product titer (mmol/L) | 6.63553±0.63057 | 6.30898±0.55137 | 19.92473±0.49566 | 8.65782±0.74377 |
| Product titer (g/L) | 3.24106±0.308 | 3.08155±0.26931 | 9.73203±0.2421 | 4.22883±0.36329 |
| Volumetric productivity (g/L/day) | 0.64821±0.0616 | 0.61631±0.05386 | 1.94641±0.04842 | 0.84577±0.07266 |
| Substrate-based product yield (g/g (Lactose)) | 1.15752±0.11 | 1.10056±0.09618 | 3.47573±0.08646 | 1.51029±0.12975 |

**Table S3.** Product parameter of surfactin in *B. subtilis* and *B. subtilis*@CdS

|  | *B. subtilis* | | *B. subtilis*@CdS | |
| --- | --- | --- | --- | --- |
|  | Light | Dark | Light | Dark |
| Fermentation time (h) | 120 | 120 | 120 | 120 |
| Product titer (g/L) | 0.006421±1.52753*10^-5^ | 0.22659±0.03094 | 0.37497±0.01023 | 0.31584±0.00492 |
| Volumetric productivity (g/L/day) | 0.00128±3.05505*10^-6^ | 0.04532±0.00619 | 0.07499±0.00205 | 0.06317±9.83953*10^-4^ |
| Substrate-based product yield (g/g (Glucose)) | 0.00105±4.11982*10^-6^ | 0.02023±0.00276 | 0.03348±9.13264*10^-4^ | 0.0282±4.39265*10^-4^ |

|  | *B. subtilis*@HgS | | *B. subtilis*@PbS | | *B. subtilis*@CdS-PbS-HgS | |
| --- | --- | --- | --- | --- | --- | --- |
|  | Dark | Light | Dark | Light | Dark | Light |
| Fermentation time (h) | 120 | 120 | 120 | 120 | 120 | 120 |
| Product titer (mmol/L) | 12.94151±3.99401 | 18.19712±0.56785 | 5.8237±0.33754 | 12.09964±1.41241 | 3.51384±0.90477 | 13.17394±3.54823 |
| Product titer (g/L) | 6.32115±1.95083 | 8.8882±0.27736 | 2.84453±0.16487 | 5.90995±0.68988 | 1.7163±0.44192 | 6.43468±1.7331 |
| Volumetric productivity (g/L/day) | 1.26423±0.39017 | 1.77764±0.05547 | 0.56891±0.03297 | 1.18199±0.13798 | 0.34326±0.08838 | 1.28694±0.34662 |
| Substrate-based product yield (g/g (Lactose)) | 2.25755±0.69673 | 3.17436±0.09906 | 1.0159±0.05888 | 2.1107±0.24638 | 0.61296±0.15783 | 2.2981±0.61896 |

**Table S4.** Product parameter of 2'-FL in *B. subtilis*@PbS, *B. subtilis*@HgS and *B. subtilis*@CdS-HgS-PbS

**Fig. S1.** Successive knockout of genes involved in H_2_S biosynthesis (*mccB*, *metC*, and *cysK*) using the Cre-loxP system. (a) Sanger sequencing chromatogram of the genomic integration junctions (upstream and downstream) for the homologous recombination fragment and the excision of the resistance marker (Zeocin). (b) Colony PCR-based analysis of gene knockout verification was resolved by agarose gel electrophoresis.

**Fig. S2.** SEM images of the non-knockout strain KO0 and the triple-gene knockout mutant KO3 strain with the presence of Cd^2+^. (a) KO0 strain exhibiting pronounced particulate deposits on the cell surface. (b) Triple-gene knockout mutant (*ΔmccB-ΔmetC-ΔcysK*) showing a few particle depositions on the cell surface.

**Fig. S3.** Effects of Cd^2+^ and L-cysteine concentrations on the growth (OD) of the strain (20-fold dilution). (a) Growth curves of the strain at different Cd^2+^ concentrations (0.1 ~ 1.0 mM) without L-cysteine, (b) Growth curves of the strain at different Cd^2+^ concentrations (0.1 ~ 1.0 mM) in the presence of 1 mM L-cysteine, (c) Growth curves of the strain at different Cd^2+^ concentrations (0.1 ~ 1.0 mM) with the addition of 10 mM L-cysteine; (d) 2’-FL production of the strain at different Cd^2+^ concentrations (0.1 ~ 1.0 mM) with the addition of 10 mM L-cysteine. The data are presented as the mean±s.d. from 3 biological replicates (n=3).

**Fig. S4.** SEM images of the strain under different concentration conditions. (a) SEM image acquired in the presence of 0.1 mM Cd^2+^ and 1 mM L-Cys, showing a smooth cell surface without obvious particulate attachments. (b) SEM image obtained with 1 mM Cd^2+^ and 1 mM L-Cys, revealing extensive cell disruption and fragmentation. (c) SEM image recorded with 1 mM Cd^2+^ and 10 mM L-Cys, showing pronounced particulate attachments on the cell surface.

**Fig. S5**. Distribution of cadmium in the culture system before and after 48 h of cultivation. Cadmium concentrations were quantified by ICP-MS in three fractions: (S1) initial culture medium immediately after Cd^2+^ addition; (S2) Supernatant after 48 h of cultivation (obtained by centrifugation); (S3) supernatant after further filtration through a 0.22-μm membrane. S1 represents the total Cd input; S2 reflects the total cadmium remaining in the liquid phase (including both dissolved Cd^2+^ and suspended CdS particles); S3 represents dissolved Cd^2+^ only, with the difference between S2 and S3 indicating the fraction of particulate CdS in the supernatant. The dramatic decrease from S1 to S2 demonstrates that the majority of Cd is associated with the cell pellet, supporting the *in situ* formation of CdS on the bacterial cell surface. The data are presented as the mean±s.d. from 3 biological replicates (n=3).

**Fig. S6**. ICP-MS quantification of cadmium in the fermentation broth supernatant before and after membrane filtration. Cadmium concentrations were measured in the cell-free supernatant obtained by centrifugation and the supernatant further filtered through a 0.22 μm membrane to remove residual particulate matter. This comparison distinguishes dissolved Cd^2^⁺ from particle-associated Cd (CdS nanoparticles) in the fermentation system.

**Fig. S7.** EDS image of CdS particles.

**Fig. S8.** The yield of 2’-FL under different wavelengths of light irradiation. The data are presented as the mean±s.d. from 3 biological replicates (n=3).

**Fig. S9.** The yield of 2’-FL under different luminous intensity of light irradiation. The data are presented as the mean±s.d. from 3 biological replicates (n=3).

**Fig. S10.** MS (ESI) of 2’-FL

**Fig. S11.** Dynamics of culture pH during the fermentation process. The pH of the fermentation broth was measured at 24-hour intervals using a pH meter (Mettler Toledo, Switzerland). The data are presented as the mean ± s.d. from 3 biological replicates (n = 3).

**Fig. S12. Live/dead staining of the pure bacterial system and the bacterial-CdS hybrid system.** Representative fluorescence microscopy images show cell viability in *B. subtilis* and *B. subtilis*@CdS. After 120 h of illumination, *B. subtilis*@CdS exhibits higher bacterial viability than the *B. subtilis-*Dark control, indicating that CdS formation and light irradiation do not cause significant inhibition of cell growth.

**Fig. S13.** Intracellular NADH/NAD⁺ ratios of *B. subtilis*@CdS after 48 h illumination. The data are presented as the mean±s.d. from 3 biological replicates (n=3).

**Fig. S14.** Rotenone inhibition of the respiratory chain demonstrates that photogenerated electrons from CdS enter the intracellular redox network. (a) 2'-FL production by *B. subtilis*@CdS under light with rotenone and light only. (b) Intracellular NADPH/NADP⁺ ratios under the same conditions as in (a). (c) Intracellular NADH/NAD⁺ ratios under the same conditions as in (a). The data are presented as the mean±s.d. from 3 biological replicates (n=3).

**Fig. S15.** MS (ESI) of surfactin

**Fig. S16.** Multi-step downstream purification strategy for Cd^2^⁺ removal from fermentation broth and ICP-MS quantification of residual cadmium in surfactin fermentation broth, respectively. T1: Fermentation broth supernatant (after centrifugation); T2: Na_2_S precipitation combined with diatomaceous earth adsorption; T3: Lewatit TP207 chelating resin ion exchange. The data are presented as the mean ± s.d. from 3 biological replicates (n = 3).

**Fig. S17. Overview of metabolite classification and sample correlation analysis based on untargeted metabolomics.** (**a**) Pie chart showing the chemical classification of detected metabolites. The metabolome is dominated by organoheterocyclic compounds (30.33%) and organic acids and derivatives (20.12%), followed by benzenoids (16.53%), lipids and lipid-like molecules (11.15%), and organic oxygen compounds (7.65%). Minor fractions include organic nitrogen compounds, nucleosides/nucleotides and analogues, alkaloids and derivatives, and other low-abundance compound classes, indicating broad metabolic coverage across central and secondary metabolism. (**b**) Heatmap of pairwise correlation coefficients among biological replicates and experimental groups. Samples within the same group (*B.Subtilis*: A1-A4 and [*B.Subtilis*@CdS](mailto:B.Subtilis@CdS): B1-B4) exhibit strong correlations (Pearson’s r ≈ 0.84 ~ 0.91), whereas correlations between different groups are substantially lower (r ≈ 0.22 ~ 0.28), demonstrating high reproducibility within groups and clear metabolic divergence between conditions.

**Fig. S18.** **KEGG pathway annotation of metabolites between *B. subtilis* and *B. subtilis*@CdS.**

Kyoto Encyclopedia of Genes and Genomes (KEGG) level-1 and level-2 pathway classification of annotated metabolites, with the y-axis indicating the number of metabolites assigned to each pathway category. At KEGG level 1, the Metabolism category overwhelmingly dominates, whereas Environmental Information Processing, Genetic Information Processing, Cellular Processes, and Organismal Systems account for comparatively minor fractions. At KEGG level 2, Global and overview maps represent the most enriched category (281 metabolites), far exceeding all other pathways, reflecting a global restructuring of intracellular metabolic fluxes rather than localized pathway perturbations. Core metabolic pathways associated with carbon utilization, reducing power generation, and biosynthetic precursor supply are prominently enriched, including amino acid metabolism (104 metabolites), carbohydrate metabolism (64 metabolites), and energy metabolism (26 metabolites). Pathways involved in cofactor and vitamin metabolism (36 metabolites) and lipid metabolism (44 metabolites) are also substantially represented, highlighting coordinated regulation of redox balance and anabolic capacity. In contrast, pathways related to genetic information processing, such as translation (16 metabolites), folding, sorting and degradation (10 metabolites), and replication and repair (1 metabolite), exhibit relatively low metabolite representation, indicating limited allocation of cellular resources toward biomass accumulation. Collectively, these results demonstrate a metabolism-focused reprogramming characterized by the redirection of carbon flux, energy, and reducing equivalents toward biosynthetic pathways, consistent with a production-oriented metabolic state promoted by light-driven electron input.

**Fig. S19. Chemical structural classification of annotated metabolites between *B. subtilis* and *B. subtilis*@CdS.**

Chemical structural classification (based on Human Metabolome Database (HMDB)) of annotated metabolites, with the y-axis indicating the number of metabolites assigned to each chemical class. The distribution reveals that the detected metabolites are predominantly enriched in organic acids and derivatives (278 metabolites), organoheterocyclic compounds (251 metabolites), lipids and lipid-like molecules (165 metabolites), and benzenoids (139 metabolites), collectively accounting for the majority of the metabolome. These metabolite classes are closely associated with central carbon metabolism, redox reactions, membrane structure, and biosynthetic precursor pools, indicating extensive remodeling of core metabolic networks. A substantial number of organic oxygen compounds (96 metabolites), phenylpropanoids and polyketides (83 metabolites), and nucleosides, nucleotides, and analogues (47 metabolites) were also identified, highlighting coordinated regulation of carbohydrate-derived intermediates, secondary metabolism, and nucleotide-associated processes.

**Fig. S20. Lipid metabolites and pathways strategy (LIPID MAPS) -based lipid subclass annotation of identified metabolites.**

Lipid classification of annotated metabolites based on the LIPID MAPS database, with the y-axis indicating the number of metabolites assigned to each lipid subclass. Fatty acyls (FA) constitute the most abundant lipid category, dominated by fatty acids and conjugates (25 metabolites), followed by fatty amides (4 metabolites) and fatty esters (3 metabolites), indicating active remodeling of fatty acid pools and carbon storage–related intermediates. Glycerophospholipids (GP) are broadly represented, including glycerophosphates (5 metabolites), glycerophosphocholines (4 metabolites), glycerophosphoethanolamines (5 metabolites), glycerophosphoglycerols (4 metabolites), and glycerophosphoinositols (1 metabolite), reflecting dynamic regulation of membrane lipid composition and phospholipid turnover. Polyketides (PK) form another prominent class, alongside aromatic polyketides (3 metabolites), angucyclines (1 metabolite), and cyclic polyketides (1 metabolite), suggesting activation or accumulation of secondary metabolite–related lipid-derived compounds. In contrast, prenol lipids (PR), including phenolic lipids (6 metabolites), isoprenoids (1 metabolite), and quinones and hydroquinones (1 metabolite), are present at relatively lower abundance. Sphingolipids (SP) are sparsely detected, including phosphosphingolipids (2 metabolites) and sphingoid bases (2 metabolites). Collectively, the LIPID MAPS annotation indicates that lipidomic alterations in the biohybrid system are primarily centered on fatty acid metabolism and glycerophospholipid remodeling, rather than sterol or sphingolipid pathways. This lipid profile is consistent with a metabolism-oriented cellular state, supporting enhanced membrane-associated electron transport, redox balancing, and precursor allocation required for efficient light-driven biosynthesis.

**Fig. S21. Differential metabolite analysis and KEGG pathway enrichment of the biohybrid system.** (**a**) Volcano plot of differentially expressed metabolites (DEMs) between the biohybrid system under illumination and the corresponding control condition. The x-axis represents log_2_foldchange, and the y-axis represents -log_10_(p value). Each point corresponds to an individual metabolite. Metabolites with significantly increased abundance are shown in red (upregulated, n = 785), whereas those with decreased abundance are shown in blue (downregulated, n = 796); non-significant metabolites are shown in gray. Dashed horizontal and vertical lines indicate the statistical significance threshold and fold-change cutoff, respectively. The large number of DEMs with substantial fold changes indicates that light-driven semiconductor biohybridization induces a global metabolic remodeling rather than limited perturbations of isolated pathways. (**b**) KEGG pathway enrichment analysis of differentially expressed metabolites. Each bubble represents an enriched KEGG pathway; the x-axis denotes the rich factor (ratio of DEMs to total metabolites annotated in a given pathway), bubble size reflects the number of enriched metabolites (Count), and color indicates the corresponding p value. Pathways with high rich factors and large metabolite counts are predominantly associated with metabolism, including microbial metabolism in diverse environments, degradation of aromatic compounds, purine metabolism, carbohydrate transport and utilization, fructose and mannose metabolism, cysteine and methionine metabolism. In contrast, pathways related to cellular regulation or signaling are less prominently enriched. The enrichment pattern demonstrates that differential metabolites are preferentially concentrated in central carbon metabolism, amino acid metabolism, and redox-related pathways, supporting the conclusion that photoelectron input drives a metabolism-centered reprogramming toward biosynthetic and catabolic optimization rather than growth-associated regulation.

**Fig. S22. KEGG pathway enrichment classification and differential abundance analysis of metabolites.** (**a**) KEGG pathway enrichment classification bar chart.

The bar chart summarizes the number of differentially expressed metabolites enriched in KEGG pathways, grouped by KEGG functional classes (Metabolism, Cellular Processes, and Organismal Systems). The x-axis indicates the metabolite count assigned to each pathway. Metabolism-related pathways are overwhelmingly enriched compared to other classes. Notably, microbial metabolism in diverse environments shows the highest enrichment (50 metabolites), followed by purine metabolism (16 metabolites), degradation of aromatic compounds (12 metabolites), and cysteine and methionine metabolism (11 metabolites). Additional enrichment is observed in carbohydrate-related pathways, including fructose and mannose metabolism (7 metabolites) and carbohydrate digestion and absorption (5 metabolites). In contrast, pathways associated with cellular processes or organismal systems, such as quorum sensing, efferocytosis, thermogenesis, and photosynthesis, contain relatively few metabolites (≤5), indicating that the metabolic response is strongly centered on core biochemical processes rather than growth regulation or signaling. (**b**) KEGG differential abundance score plot. This plot displays the differential abundance score of enriched KEGG pathways, reflecting the overall direction and magnitude of metabolite changes within each pathway. Positive scores indicate pathways dominated by upregulated metabolites, whereas negative scores indicate pathways dominated by downregulated metabolites. Several metabolism-centered pathways, including fructose and mannose metabolism, cysteine and methionine metabolism, purine metabolism, microbial metabolism in diverse environments, and degradation of aromatic compounds, exhibit pronounced differential abundance with high statistical significance. Bubble size represents the number of metabolites involved, and color denotes the p value, indicating that pathways with larger metabolite coverage tend to show stronger and more coordinated metabolic shifts. Overall, the differential abundance analysis highlights a global metabolic reprogramming characterized by pathway-level coordination rather than isolated metabolite changes.

**Fig. S23.** (a) SEM image of *E. coli*@CdS. (b) EDS spectrum from *E. coli*@CdS biohybrid.

**References**

1. H. THOMAS, “Conversion of Methionine to cysteine in *Bacillus subtilis* and its regulation,” *J. Bacteriol.* *189*, (2007): 187-197 .
2. P. Yan, J. Li, J. Wu, Q. He, X. Wang, X. Wang, Y. Liang, Z. Li, “Cre/lox system and PCR-based genome engineering in *Bacillus subtilis*,” *Appl. Environ. Microbiol*. 74, (2008): 5556-5562.
